# *Akkermansia muciniphila* in neuropsychiatric disorders: friend or foe?

**DOI:** 10.3389/fcimb.2023.1224155

**Published:** 2023-07-10

**Authors:** Wenhui Lei, Yiwen Cheng, Jie Gao, Xia Liu, Li Shao, Qingming Kong, Nengneng Zheng, Zongxin Ling, Weiming Hu

**Affiliations:** ^1^ Jinan Microecological Biomedicine Shandong Laboratory, Shandong First Medical University, Jinan, Shandong, China; ^2^ Jinan Microecological Biomedicine Shandong Laboratory, Jinan, Shandong, China; ^3^ Collaborative Innovation Center for Diagnosis and Treatment of Infectious Diseases, State Key Laboratory for Diagnosis and Treatment of Infectious Diseases, National Clinical Research Center for Infectious Diseases, The First Affiliated Hospital, School of Medicine, Zhejiang University, Hangzhou, Zhejiang, China; ^4^ Department of Intensive Care Unit, The First Affiliated Hospital, School of Medicine, Zhejiang University, Hangzhou, Zhejiang, China; ^5^ School of Clinical Medicine, Institute of Hepatology and Metabolic Diseases, The Affiliated Hospital of Hangzhou Normal University, Hangzhou Normal University, Hangzhou, Zhejiang, China; ^6^ School of Biological Engineering, Hangzhou Medical College, Institute of Parasitic Diseases, Hangzhou, Zhejiang, China; ^7^ Department of Obstetrics, The First Affiliated Hospital, School of Medicine, Zhejiang University, Hangzhou, Zhejiang, China; ^8^ Department of Psychiatry, Quzhou Third Hospital, Quzhou, Zhejiang, China

**Keywords:** Akkermansia muciniphila, Alzheimer’s disease, depression, microbiota-gut-brain axis, neuropsychiatric disorders

## Abstract

An accumulating body of evidence suggests that the bacterium *Akkermansia muciniphila* exhibits positive systemic effects on host health, mainly by improving immunological and metabolic functions, and it is therefore regarded as a promising potential probiotic. Recent clinical and preclinical studies have shown that *A. muciniphila* plays a vital role in a variety of neuropsychiatric disorders by influencing the host brain through the microbiota-gut-brain axis (MGBA). Numerous studies observed that *A. muciniphila* and its metabolic substances can effectively improve the symptoms of neuropsychiatric disorders by restoring the gut microbiota, reestablishing the integrity of the gut mucosal barrier, regulating host immunity, and modulating gut and neuroinflammation. However, *A. muciniphila* was also reported to participate in the development of neuropsychiatric disorders by aggravating inflammation and influencing mucus production. Therefore, the exact mechanism of action of *A. muciniphila* remains much controversial. This review summarizes the proposed roles and mechanisms of *A. muciniphila* in various neurological and psychiatric disorders such as depression, anxiety, Parkinson’s disease, Alzheimer’s disease, multiple sclerosis, strokes, and autism spectrum disorders, and provides insights into the potential therapeutic application of *A. muciniphila* for the treatment of these conditions.

## 1 Introduction

The gut microbiota plays a crucial role in maintaining host health and was once regarded as a “forgotten organ” (Cheng et al., 2020; Ling ZX. et al., 2022). A large number of commensal bacteria, viruses, fungi, parasites, and archaea thrive both within and on the human body and participate in human physiology, growth, development, immunity, nutrition, and metabolism. Recent advances in microbiology, particularly through the application of multi-omics approaches, have revealed that the gut microbiota transcends its previous characterization as a passive bystander and actively impacts host health and disease. Despite being previously overlooked, the role of the gut microbiota as a key contributor to the pathology of various host diseases is now gaining recognition. Mounting evidence from clinical and preclinical studies suggests that gut eubiosis is critical for host health, while gut dysbiosis disturbs gut homeostasis and contributes to the onset of host diseases. The intimate interplay between the gut microbiota and the host has revitalized our current understanding of disease pathogenesis, proposing novel targets for disease diagnosis and therapeutic interventions. Within the field of microbiota research, a considerable number of crucial functional microorganisms have been identified. Ongoing efforts to decipher the underlying roles and mechanisms of these microorganisms will contribute to our understanding of their functional impact on host health and disease.

Recently, *A. muciniphila* has received significant attention in scientific research. *A. muciniphila* is the sole representative of the phylum *Verrucomicrobia* in the human gut and is a normal component of its microbiota that is widely distributed in the mucus layer (Derrien et al., 2004). This strictly anaerobic gram-negative bacterium possesses oval non-motile cells and cannot produce endospores. *A. muciniphila* can use mucins as its sole nitrogen, carbon, and energy source and forms white colonies on soft agar mucin medium (Derrien et al., 2004). In healthy individuals, *A. muciniphila* accounts for approximately 3–5% of the gut microbiota (Belzer and De Vos, 2012). Qin et al. observed that *A. muciniphila* was among the top 20 most abundant species in the human gut (Qin et al., 2010). However, the colonization and abundance of *A. muciniphila* may be influenced by various confounding factors such as age, location, genotype, dietary habits, and physiological status of the host (Zhai et al., 2019). *A. muciniphila* is present in different parts of the human mucosa and feces, but is most abundant in the mucin-producing cecum (Derrien et al., 2011). Recent studies conducted on participants of different nationalities have shown that the abundance of *A. muciniphila* changes with host age (Collado et al., 2007; Cheng et al., 2016). *A. muciniphila* demonstrates stable colonization within the human gastrointestinal tract (GIT) during the first year of life, reaching similar levels as those found in healthy adults. However, a noticeable decline in its abundance is observed in older adults (Collado et al., 2007). These changes may be associated with the alterations in mucus quality and quantity that occur during aging. *A. muciniphila* exclusively degrades mucin and is equipped with an active acid-resistance system, allowing it to degrade human milk oligosaccharides within the stomachs of newborn infants, contributing to improved mucosal and metabolic health later in life (Bosscher et al., 2001; Kostopoulos et al., 2020).

Over the past decade, *A. muciniphila* has been increasingly investigated and recognized as a true gut symbiont that promotes beneficial interactions in the human GIT. *A. muciniphila* plays an important role in modulating glucose tolerance, energy metabolism, and the maturation and functioning of the immune system. *A. muciniphila* is currently a popular research subject due to its potential beneficial impact on various health conditions such as overweight, diabetes, obesity, hypertension, colitis, and age-related complications (Dao et al., 2016; Li et al., 2017; Liu R. et al., 2017; Plovier et al., 2017; Grander et al., 2018; Bian et al., 2019; Depommier et al., 2019; Payahoo et al., 2019; Grajeda-Iglesias et al., 2021; Liu et al., 2021). The etiology of these conditions may stem from dysregulation in the gut microbiota, intestinal mucosal damage, and high permeability of the intestinal mucosa during the early stages of development. *A. muciniphila* contributes to the restoration of the gut microbiota and maintenance of a healthy gut mucosal barrier, thereby regulating gut immunity and limiting the onset of inflammation (Cani et al., 2022). Van der Lugt et al. showed that supplementation with *A. muciniphila* can increase the thickness of the colonic mucus layer by nearly threefold (Van Der Lugt et al., 2019). Zhao et al. also demonstrated that *A. muciniphila* can improve chronic low-grade inflammation by increasing anti-inflammatory factors, such as α‐tocopherol, and reducing levels of lipopolysaccharide (LPS)-binding protein (Zhao et al., 2017). Another study found that *A. muciniphila* can increase the amount of 2-oleoylglycerol in the gut and stimulate the production of glucagon-like peptide-1 (GLP-1) in L-cells. GLP-1 is known for its beneficial effects, including appetite suppression, anti-diabetic properties, and weight loss (Everard et al., 2013). *A. muciniphila* secretes a GLP-1-inducing protein that can improve glucose homeostasis and ameliorate metabolic diseases in high-fat diet (HFD)-fed C57BL/6J mice (Yoon et al., 2021). Furthermore, Plovier et al. showed that supplementation with *A. muciniphila* or its protein extract significantly reduced plasma high-density lipoprotein (HDL) cholesterol concentrations and reversed HFD-induced hypercholesterolemia (Plovier et al., 2017). Everard et al. demonstrated that treatment with viable *A. muciniphila* could reverse HFD-induced metabolic disorders, including metabolic endotoxemia, fat mass gain, insulin resistance, and adipose tissue inflammation (Everard et al., 2013). Greer et al. revealed that *A. muciniphila* mediates the negative effects of interferon-gamma (IFN-γ) on glucose metabolism, highlighting its significant role in promoting metabolic health (Greer et al., 2016). Moreover, *A. muciniphila* induces adaptive immune responses in the GIT, contributing to the maintenance of GIT homeostasis (Ansaldo et al., 2019). The increased endocannabinoids induced by *A. muciniphila* can play a role in controlling GIT inflammation, promote gut mucus production, and enhance the expression of antimicrobial peptides such as regenerating islet-derived 3-gamma (Reg3γ), thereby supporting innate immunity (Everard et al., 2013). Derrien et al. observed that *A. muciniphila* modulates the pathways involved in establishing homeostasis for basal metabolism and immune tolerance of the gut commensal microbiota (Derrien et al., 2011). Through the activation of the non-canonical Toll-like receptor (TLR) heterodimer, TLR2-TLR1, by a phospholipid agonist, *A. muciniphila* selectively stimulates proinflammatory cytokines, thereby promoting homeostatic host immune responses (Bae et al., 2022). A recent study by Liu et al. found that *A. muciniphila* can also induce immune responses mediated by RORγt^+^ regulatory T cells, leading to the amelioration of dextran sulfate sodium (DSS)-induced colitis (Liu Y. et al., 2022). In addition, *A. muciniphila* has shown efficacy in improving obesity, hepatic steatosis, type 1 and 2 diabetes mellitus (T1DM and T2DM), and intestinal inflammation in mice (Cani et al., 2022). Currently, *A. muciniphila* is considered a promising probiotic candidate for the prevention and treatment of human metabolic diseases, inflammatory disorders, and other microbiota-related diseases; however, its potential adverse effects need to be further investigated as several studies have also observed the harmful effects of *A. muciniphila* on human host (Ganesh et al., 2013; Weir et al., 2013; Cekanaviciute et al., 2017; Wang F. et al., 2022).

The positive effects of *A. muciniphila* are not limited to the improvement of metabolic functions and modulation of the immune system. Recent studies have shown that *A. muciniphila* actively participates in various neurological and psychiatric disorders such as depression and anxiety (Ding et al., 2021; Sun et al., 2023), multiple sclerosis (MS) (Berer et al., 2017; Ling et al., 2020a), Alzheimer’s disease (AD) (Ling et al., 2020b; Ou et al., 2020), Parkinson’s disease (PD) (Fang et al., 2021), autism spectrum disorders (ASDs) (Goo et al., 2020), and strokes (Stanley et al., 2018). Accumulating evidence suggests that the effects of *A. muciniphila* on these conditions are most likely mediated by the gut-brain axis (GBA). The GBA refers to the bidirectional communication between the gut and brain, representing the connection between the GIT and the central nervous system (CNS) (Grenham et al., 2011; Sherwin et al., 2016). The GBA plays a crucial role in maintaining and coordinating GIT function while also influencing mood, motivated behavior, and higher cognitive functions through feedback from the gut (Foster et al., 2017). In addition to this, the gut microbiota can directly produce metabolites and immunomodulatory factors that directly communicate with the CNS to regulate brain function (Sherwin et al., 2016). This review focuses on the role of *A. muciniphila* in various neuropsychiatric disorders, explores the possible underlying mechanisms behind its involvement, and discusses its therapeutic potential through GBA modulation.

## 2 Underlying mechanisms of *A. muciniphila* in neuropsychiatric disorders

*A. muciniphila* is a potential next-generation probiotic that plays an important role in systemic metabolism, intestinal health, and immune regulation (Zhao et al., 2023). However, limited information is available on the direct effects of *A. muciniphila* on neuropsychiatric disorders, as the majority of current clinical studies have primarily focused on exploring the association between the bacterium and the disorders without establishing causation. Nevertheless, several hypotheses explaining the mechanisms underlying the impact of *A. muciniphila* on brain function have been proposed. These hypotheses involve the production of neuroactive metabolites and derivatives, protection of the GIT mucosal barrier, regulation of host immunity, and modulation of metabolism.

### 2.1 Neuroactive metabolites and derivatives

*A. muciniphila* exhibits the ability to generate various neuroactive metabolites, including short-chain fatty acids (SCFAs), B-vitamins, kynurenic acid, and γ-aminobutyric acid (GABA), through the breakdown of mucins or interactions with other beneficial microbes that play vital roles in the occurrence of neuropsychiatric disorders. Amuc_1100, the outer membrane protein of *A. muciniphila*, likely plays a crucial role in the interaction of the bacterium with its host and can restore the function of the GIT mucosal barrier and promote intestinal 5-hydroxytryptamine (5-HT) biosynthesis through TLR2 signaling. A recent study identified P9, an 84 kDa protein, that stimulates the secretion of GLP-1 (Yoon et al., 2021). In addition, *A. muciniphila*-derived extracellular vesicles (AmEVs) serve as potent vehicles for intercellular communication, facilitating the normalization of the gut microbiota, improvement of GIT permeability, and modulation of inflammatory responses. Collectively, these findings establish *A. muciniphila* as a neuroactive microbe.

#### 2.1.1 SCFAs

SCFAs are exclusively produced by microbial fermentation in the host and are regarded as indispensable signals in the communication between the gut and extraintestinal organs. *A. muciniphila* utilizes mucins to generate a diverse range of SCFAs, such as butyrate, acetate, and propionate. These SCFAs play a vital role in preserving gut health and have been associated with various health benefits, including immunoregulation and the protection of intestinal barrier integrity. Recent studies have suggested a possible connection between *A. muciniphila*-produced SCFAs and neuropsychiatric disorders, as *A. muciniphila* can directly alter bacterial composition and SCFAs production in the gut (Xia et al., 2022). Research has shown that individuals with depression and anxiety tend to have lower levels of certain types of SCFAs in their gut and that probiotics containing *A. muciniphila* can help increase the production of these SCFAs (Li et al., 2022). Furthermore, preliminary findings suggest a link between *A. muciniphila* and neuropsychiatric disorders like ASD, schizophrenia, and PD, although more research is required to fully understand this connection. Studies have found that high concentrations of butyrate rapidly increase histone acetylation, leading to improved learning and memory, reduced depression and persistent behavior, and improved social skills in mouse models with ASD (De Theije et al., 2014; Kratsman et al., 2016). A recent clinical trial revealed that oral supplementation with butyrate can enhance cognitive performance and reduce negative symptoms in drug-naïve patients with first-episode schizophrenia (Li et al., 2021). In mice, SCFAs improved schizophrenia-like symptoms, including social withdrawal, altered sensorimotor gating, and working memory deficits (Joseph et al., 2017; Dalile et al., 2019). In a PD mouse model, butyrate was found to improve motor impairment, alleviate dopaminergic neuronal degeneration, and inhibit neuroinflammation (Hou et al., 2021). Recent studies have shown that GLP-1 has the ability to influence brain function by improving movement disorders, alleviating inflammatory responses and oxidative stress, and inhibiting apoptosis (Chen et al., 2018; Batista et al., 2019). *A. muciniphila*-produced SCFAs specifically activate the expression of G-protein-coupled receptors in enteroendocrine L-cells in the distal small intestine and colon, regulating the production of GLP-1 and other hormones (Psichas et al., 2015). The available evidence suggests that *A. muciniphila*-produced SCFAs also possess direct neuropsychiatric effects by modulating the activity of the enteric nervous system (ENS) and the vagus nerve, crossing the blood-brain barrier (BBB) and directly affecting the neurotransmitter and neuropeptide systems in the brain (Borre et al., 2014; Vicentini et al., 2021).

The mechanisms underlying the impact of *A. muciniphila*-produced SCFAs on neuropsychiatric disorders are complex. Based on the results of preclinical and clinical studies, several hypotheses have emerged. First, SCFAs may modulate the GBA, which has a significant role in the regulation of mood, cognition, and behavior. Animal studies have demonstrated that supplementation with certain SCFAs can improve anxiety- and depression-like behaviors, reduce stress-induced neuroinflammation, and even increase the production of neurotransmitters such as serotonin and GABA, which are implicated in neuropsychiatric disorders. Second, SCFAs contribute to the reduction of systemic inflammation, which is strongly associated with the development of neuropsychiatric disorders (Yao et al., 2022). For example, patients with depression often exhibit elevated levels of inflammatory markers like C-reactive protein and IL-6, and anti-inflammatory drugs can improve depressive symptoms (Jiang et al., 2015). SCFAs have potent anti-inflammatory effects and may reduce inflammation in the CNS and gut. Finally, the beneficial effects of *A. muciniphila*-produced SCFAs on neuropsychiatric disorders may be mediated by other mechanisms, such as changes in gene expression, epigenetic modifications, or gut microbiota modulation. Considering the complexity of the GBA and the gut microbiota, it is likely that multiple mechanisms are involved. Overall, the exact mechanisms underlying the neuropsychiatric effects of *A. muciniphila*-produced SCFAs are still under investigation. However, the impact of these SCFAs on brain function presents a promising therapeutic target for the treatment of neuropsychiatric disorders.

#### 2.1.2 Amuc_1100 and P9

Amuc_1100, a thermostable outer membrane protein specific to *A. muciniphila*, is speculated to play an important role in the intricate interaction between microbes and hosts, reproducing the beneficial effects of *A. muciniphila* (Anhe and Marette, 2017). The absence of mucin induces the expression of the gene encoding Amuc_1100 (Shin et al., 2019). Amuc_1100 can activate TLR2-mediated signaling pathways which contribute to the enhancement of gut barrier integrity and the restoration of tight junction expression (Plovier et al., 2017). Recent studies have implicated Amuc_1100 in the modulation of brain function and behavior. As previously mentioned, Wang et al. discovered that Amuc_1100, through the TLR2 signaling pathway, upregulates the rate-limiting enzyme tryptophan hydroxylase 1 (Tph1), resulting in increased expression of 5-HT in the GIT and downregulation of the expression of the serotonin transporter (SERT) (Wang et al., 2021). 5-HT may improve GIT function and nutrition, and disturbances in the physiological function of the GIT have been associated with various mental health disorders, including ASD (Ferguson et al., 2017). 5-HT is a key regulator of the development and function of the ENS and CNS, and is likely able to connect the microbiota-gut-brain nexus. An insufficient amount of 5-HT may lead to the development of depression and other neuropsychiatric disorders. Recent findings have demonstrated the antidepressant effects of Amuc_1100, which improves dysregulated gut microbiota, increases the levels of brain-derived neurotrophic factor (BDNF), and inhibits inflammation in the hippocampus induced by chronic unpredictable mild stress (CUMS) (Cheng R. et al., 2021). In addition, Amuc_1100 alleviates antibiotic-induced anxiety and depression by modulating the BDNF/tropomyosin receptor kinase B (BDNF/TrkB) signaling pathway, thereby restoring the levels of BDNF and TrkB in the hippocampus and cortex (Sun et al., 2023). Amuc_1100^Δ80^, a truncated form of Amuc_1100 lacking the first 80 N-terminal amino acids, exhibits a higher affinity for TLR2 compared to the wild-type protein. Amuc_1100^Δ80^ can affect the level of 5-HT and the downstream 5-HTR1A-CREB-BDNF signaling pathway through its interaction with TLR2 and the modulation of the gut microbial composition, leading to a superior antidepressant effect compared to Amuc_1100 in ameliorating CUMS-induced depression in mice (Cheng et al., 2022). These studies suggest that Amuc_1100 or Amuc_1100^Δ80^ can modulate brain function and behavior, providing a foundation for the development of new therapeutic strategies. However, further investigations are necessary to fully understand the mechanisms responsible for these effects.

Yoon et al. recently discovered P9, an 84 kDa protein secreted by *A. muciniphila*. Their study revealed that oral administration of P9 substantially improved glucose homeostasis in mice (Yoon et al., 2021). Notably, P9 can bind to intercellular adhesion molecule-2 (ICAM-2) and activate phospholipase C (PLC), leading to intracellular Ca^2+^ signaling and cAMP response element-binding protein (CREB) activation (Si et al., 2022). Furthermore, purified P9 on its own is sufficient to trigger the production of GLP-1 by L-cells. P9-stimulated IL-6 expression in macrophages is involved in GLP-1 secretion, whereas IL-6 deficiency replicates the effects of P9 on glucose homeostasis while downregulating the expression of ICAM-2. This demonstrates that the anti-obesogenic effects are dependent on IL-6 (Yoon et al., 2021). Intestinal GLP-1 affects numerous neuronal processes. It can modulate activity in hippocampal circuits, stimulate neurite outgrowth, promote cell survival, and upregulate the production of enzymes and neurotransmitters (Holst et al., 2011). Evidence suggests that *A. muciniphila* P9 is involved in the regulation of brain function and behavior. Similar to Amuc_1100, P9 has been found to possess immunometabolic and immunomodulatory functions. Initial investigations have indicated that P9 may play a crucial role in balancing proinflammatory and anti-inflammatory responses in the GIT, leading to a decrease in the production of proinflammatory cytokines and an increase in the production of anti-inflammatory cytokines (Zheng et al., 2022). Nevertheless, the precise roles and underlying mechanisms of P9 in brain function and behavior remain unknown. Further studies are required to fully understand the mechanisms involved and to explore the potential clinical applications of *A. muciniphila* P9 in neuropsychiatric disorders.

#### 2.1.3 AmEVs

Extracellular vesicles (EVs) are submicron-sized lipid bilayer structures secreted by the gut microbiota (Ellis and Kuehn, 2010). Recent evidence suggests that bacteria-derived EVs are able to transfer genetic material and proteins from the bacteria to the host, demonstrating their diverse roles in the microbial community (Kuehn and Kesty, 2005). EVs can be considered as functional units of the gut microbiota that mediate the interaction between the host and microbiota by directly interacting with epithelial and immune cells to initiate various signaling pathways. AmEVs are small membrane-bound particles released by *A. muciniphila* and contain a variety of biomolecules, including lipids, proteins, and nucleic acids (Kim YS. et al., 2013). Chelakkot et al. observed a higher amount of AmEVs in the feces of healthy individuals compared to those with T2DM (Chelakkot et al., 2018). Furthermore, administration of AmEVs resulted in enhanced tight junction function, as evidenced by increased occluding expression. This treatment also improved glucose tolerance and reduced weight gain in HFD-induced diabetic mice, whereas EVs derived from *Escherichia coli* did not exhibit similar effects (Chelakkot et al., 2018). These findings suggest that AmEVs may serve as functional units involved in the regulation of gut permeability. By promoting the integrity of the intestinal mucosal barrier, metabolic functions in HFD-fed mice can be improved. In addition, AmEVs restored the gut microbiota composition, improved the integrity of the intestinal mucosal barrier, regulated inflammatory responses, and subsequently prevented liver injury in mice administered with HFD/CCl4 (Keshavarz Azizi Raftar et al., 2021). AmEVs decrease the expression of proinflammatory cytokines (IL-6 and TNF-α) and the LPS recognition marker TLR-4 (Alhawi et al., 2009), and they also activate TLR-2 to induce the expression of anti-inflammatory factors, suggesting that they play an important role in host anti-inflammatory responses (Ashrafian et al., 2019). In addition, AmEVs increase levels of 5-HT in the colon and hippocampus (Yaghoubfar et al., 2020). Considering their impact on the gut microbiota, gut mucosal barrier, and immunoregulation, AmEVs may also hold considerable potential in the treatment of neuropsychiatric disorders. This is a promising area of research in the field of gut microbiota-based therapeutics for neuropsychiatric disorders. However, to fully grasp the therapeutic potential of AmEVs and ensure their safe and effective utilization in treating neuropsychiatric disorders, further studies are necessary.

### 2.2 Gut mucosal barrier protection

Dysfunction of the gut mucosal barrier contributes to the development of several neuropsychiatric disorders, such as depression, anxiety, and ASD. The gut mucosal barrier is the interface between the GIT microbes and host tissue, and it plays a key role in maintaining a balanced response between the host and its microbiota (Konig et al., 2016). Preserving the integrity of the gut epithelial barrier is of utmost importance for maintaining overall host health, as it is serves as a key defense mechanism against pathogens. Previous research has demonstrated the significant role of *A. muciniphila* in the mutualistic relationship between the gut microbiota and the host, particularly in manipulating the function of the gut mucosal barrier as well as other physiological and homeostatic processes associated with obesity and T2DM (Everard et al., 2013). *A. muciniphila* primarily resides in the mucus layer of the large intestine where it regulates the proliferation of intestinal epithelial cells through the Wnt/β-catenin signaling pathway, thereby facilitating the repair of the gut mucosal barrier *in vivo* (Ring et al., 2014). Reunanen et al. found that *A. muciniphila* can improve the integrity of intestinal cells by forming strong associations with the cultured colony epithelial cell lines, Caco-2 and HT-29 (Reunanen et al., 2015). Furthermore, *A. muciniphila* has been shown to mitigate damage to the gut mucosal barrier in recipient mice by upregulating the expression of mucin 2 and increasing the number of goblet cells and mucin 2-positive cells in each villus (Chen et al., 2021). The mucus layer, primarily composed of mucin 2 secreted by goblet cells, serves as the initial physical barrier, effectively shielding the colon from toxins and pathogenic microbes (Johansson et al., 2011; Ambort et al., 2012). Mucin 2 plays a key role in maintaining a suitable distance between the GIT microbes and the epithelial surface. Aberrant expression of mucins is observed in various gastrointestinal pathologies, including infections and inflammation (Breugelmans et al., 2022). In children with ASD and their siblings, a lower number of *A. muciniphila* was found to be positively correlated with a thinner GIT mucus barrier (De Magistris et al., 2010). Despite being recognized as a mucin degrader, the ability of *A. muciniphila* to increase the thickness of the mucus layer has been previously reported (Everard et al., 2013; Li et al., 2016). The observed increase in mucus layer thickness following supplementation with *A. muciniphila* suggests that this bacterium can actively stimulate host colonic mucus production (Derrien et al., 2017). Chen et al. demonstrated that the administration of *A. muciniphila*, by restoring colonic mucus and modifying the gut microbiota, could protect against the aggravation of colitis and gut microbiota-mediated colonic mucosal barrier damage induced by psychological disorders (Chen et al., 2021). Yu et al. observed that *A. muciniphila* ameliorated the disruption of the GIT mucosal barrier caused by high levels of fructose and restraint stress through maintaining normal secretion of antimicrobial peptides in Paneth cells, promotion of tight junction protein expression, and inhibition of proinflammatory cytokines expression (Yu et al., 2022). The expression of tight junction proteins affects the permeability of the GIT mucosal barrier (Feldman et al., 2005). Chelakkot et al. demonstrated that AmEVs increased the levels of tight junction proteins and activated AMPK (Chelakkot et al., 2018). AMPK is involved in tight junction regulation by assembling tight junction proteins at cell-cell junctions (Zheng et al., 2007). Additionally, Amuc_1100 was recently found to promote gut barrier integrity by upregulating the expression of tight junction proteins (Anhe and Marette, 2017). Previous studies have demonstrated the ability of *A. muciniphila* to degrade the mucus layer, raising concerns about its potential pathogenicity in the gut mucosal barrier. However, unlike gut pathogens, *A. muciniphila* primarily resides in the outer mucosal layer and does not invade the inner one, which is a crucial requirement for pathogenicity (Tuomola et al., 2001; Derrien et al., 2010; Gómez-Gallego et al., 2016; Zhang et al., 2019). Overall, these findings suggest that *A. muciniphila* plays an important role in maintaining and protecting the mucosal barrier in the GIT.

### 2.3 Immune regulation

In addition to maintaining and protecting the gut mucosal barrier, *A. muciniphila* also plays an important role in regulating the GIT immune system. Inflammation causes damage to the CNS in both neonates and adults (Hagberg and Mallard, 2005; Degos et al., 2010; Dickson et al., 2017). *A. muciniphila* has been found to exert anti-inflammatory effects in both animal and human studies. Guo et al. demonstrated that supplementation with *A. muciniphila* reduced the levels of proinflammatory factors such as IL-1β, IL-6, and TNF-α (Guo et al., 2022). Another study showed that treatment with *A. muciniphila* can induce the synthesis of systemic and gut anti-inflammatory cytokines, while reducing the amounts of serum proinflammatory cytokines (TNF-α, IL-1α, and IL-6) and chemokines (G-CSF, MIP-1α and KC) in mice with DSS-induced colitis, thereby alleviating inflammation (Bian et al., 2019). Studies have also shown that *A. muciniphila* or its protein component, Amuc_1100, can induce the synthesis of the anti-inflammatory cytokine, IL-10, thereby improving immunological homeostasis in the GIT mucosa and enhancing gut mucosal barrier function (Ottman et al., 2017; Bian et al., 2019). Hu et al. found that the oral administration of *A. muciniphila* to H7N9-infected mice resulted in a significant reduction in pulmonary viral titers and levels of IL-6 and IL-1β. On the other hand, levels of IL-10, IFN-β and IFN-γ were enhanced, suggesting that the anti-influenza activity of *A. muciniphila* can most likely be attributed to its anti-inflammatory and immunoregulatory properties (Hu et al., 2020). Nishimura et al. demonstrated the ability of cytotoxic T-lymphocytes (CTLs) to promote the recruitment and activation of macrophages during inflammation (Nishimura et al., 2009). By reducing the number of CTLs, *A. muciniphila* and Amuc_1100 both inhibit the recruitment and activation of macrophages, most notably CD16/32^+^ macrophages (M1) (Wang et al., 2020). Since M1 macrophages can cause a rapid proinflammatory response to infection and tissue injury (Liu PS. et al., 2017; Na et al., 2019). Thus, the reduction of M1 macrophages can alleviate the inflammatory symptoms of the body, a reduction in their population can alleviate inflammatory symptoms in the body. *A. muciniphila* also affects the structure and composition of the gut microbiota, potentially reducing host proinflammatory factors and cytotoxins (Wu W. et al., 2017). While a higher relative abundance of *A. muciniphila* is associated with the development of several neuropsychiatric disorders such as AD, increased levels of *A. muciniphila* in Aβ precursor protein (APP)/presenilin (PS1) transgenic mice improved the function of the intestinal barrier and prevented the inflammatory response caused by LPS translocation (Xin et al., 2018). Furthermore, microgliosis has been implicated in the development of several neuropsychiatric disorders, and treatment with *A. muciniphila* can reduce hippocampal microglial proliferation and restore neuronal development and synaptic plasticity (Yang et al., 2019). The results from these studies suggest that *A. muciniphila* plays an important role in immune regulation and may participate in the modulation of the microbiota-gut-brain axis (MGBA).

### 2.4 Metabolic regulation

Metabolic syndromes, such as obesity and diabetes, have been associated with an increased risk of neuropsychiatric disorders, such as AD, PD, and Huntington’s disease. Both human and animal studies have been utilized to explore the concurrence of neuropsychiatric disorders and metabolic diseases, revealing shared pathophysiological mechanisms, such as dyslipidemia (Yu et al., 2020), insulin resistance (Ma et al., 2022), neurovascular dysfunction (Xu et al., 2022), neuronal cell loss (Bharadwaj et al., 2017), and tubulin-associated unit (tau) phosphorylation (Freude et al., 2005). Song et al. found that the number of patients with cognitive decline due to metabolic imbalances has increased considerably (Song, 2023). Mounting evidence suggests that metabolic regulation is necessary to prevent and treat neuropsychiatric disorders. *A. muciniphila* plays an important role in metabolic regulation, particularly in energy metabolism and glucose homeostasis. A large portion of the *A. muciniphila* genome consists of genes encoding proteins involved in metabolic processes. These include proteases, sialate esterases, and glycohydrolases (Van Passel et al., 2011). *A. muciniphila* can influence triglyceride synthesis by modulating the expression of sterol regulatory element-binding protein (SREBP) genes (Linden et al., 2018). In addition, *A. muciniphila* was found to decrease the production of serum 3β-chenodeoxycholic acid, an inhibitor of insulin secretion in T2DM patients (Zhang et al., 2021), suggesting that *A. muciniphila* may improve insulin secretion in these patients. Fujisaka et al. demonstrated that *A. muciniphila* was also able to improve gut motility, further contributing to metabolic regulation (Fujisaka et al., 2020). Therefore, *A. muciniphila* is believed to play a vital role in regulating metabolism and potentially reducing the risk of developing neuropsychiatric disorders.

## 3 *A. muciniphil*a in neuropsychiatric disorders

Accumulating evidence suggests an association between *A. muciniphila* and the development of neuropsychiatric disorders. However, the observed patterns of *A. muciniphila* have been inconsistent across different studies. The relative abundance of *A. muciniphila* varied significantly among different neuropsychiatric disorders. We have described the changes in the abundance and role of *A. muciniphila* in several neuropsychiatric disorders, including depression, AD, PD, MS, ASD, and stroke (**Table 1**).

**Table 1:** Clinical and pre-clinical findings of the alterations of *A. muciniphila* in several neuropsychiatric disorders.

Neuropsychiatric disorder	Experimental subjects	Intervention	Major findings	References
**Depression**	Social defeat stress mouse model	16S rRNA gene V3-V4 regions sequencing	•Altered gut microbiota: ↓*A. muciniphila*, *Ruminococcus*, unclassifed *Mollicutes*, *Paraprevotella*, *Dorea* ↑*Oscillospira*, *Bacteroides*, *Lachnospiraceae* •*Akkermansia* a correlate with behavioral metrics	(Mcgaughey et al., 2019)
	Sleep deprivation mouse model	16S rRNA gene V3-V4 regions sequencing	•Altered gut microbiota: •↓*A. muciniphila*, *Lactobacillus murinus* •↑ *Bacteroides massiliensis*	(Park et al., 2020)
	CRS mouse model	*A. muciniphila* (ATCC^®^ BAA-835™) 1.0×10^8^ CFU/200 µl, 3 weeks	•↓CRS−induced depressive−like behavior •↑Corticosterone; Dopamine and BDNF •Regulates gut microbiota at the phylum and genus levels •Regulate gut microbiota function •↑β-alanyl-3-methyl-l-histidine and edaravone	(Ding et al., 2021)
	Antibiotic-treated mouse model	*A. muciniphila* (1.5 × 10^9^ CFU/200 µl) Amuc-1100 (100μg/200µl) 3 weeks	•Alleviated anxiety and depression-like behaviors •Affected the gut microbiota composition •Normalized BDNF/TrkB-related genes in different brain regions •↑5-HT in serum and hippocampus •Affected the HPA axis	(Sun et al., 2023)
	Murine alcohol-LPS (mALPS) mouse model	*A. muciniphila* 1.5×10^8^ CFU/200 µl, pretreatment 2 weeks	•*A*meliorated depression-like behaviors •↑occludin, BDNF and 5-HT •↓LPS, TNF-α, IL-1β and IL-6	(Guo et al., 2022)
**AD**	25 Egypt AD patients 25 healthy controls	16S rRNA gene V3-V4 regions sequencing	•↑*Akkermansia*, *Enterobacteria*, *Bacteroidetes*, *Bacillus cereus*, *Prevotella*, *Clostridium cluster* IV •↓*Bifidobacterium*, *Firmicutes*, *Actinobacteria* spp. •Lactic acid bacteria and Prevotella were negatively correlated with cognitive impairment	(Khedr et al., 2022)
	13 Chinese mild AD patients 13 healthy controls	16S rRNA gene V3-V4 regions sequencing	•β-diversity different •↑*Akkermansia*, *Bifidobacteria* •*Bifidobacterium*, *Akkermansia*, *Coprococcus*, *Anaerostipes*, *Sutterella*, *Coprobacillus* as potential markers	(Wang Y. et al., 2022)
	100 Chinese AD patients 71 healthy controls	16S rRNA gene V3-V4 regions sequencing	•↓Bacterial diversity •↑*Akkermansia*, *Bififidobacterium* •↓*Faecalibacterium*, *Roseburia*, *Clostridium sensu stricto*, *Gemmiger*, *Dialister*, *Romboutsia*, *Coprococcus*, *Butyricicoccus* •*Faecalibacterium* was positively correlated with MMSE, WAIS, and Barthel scores	(Ling et al., 2020b)
	APPswe/PS1de9 (APP/PS1) mouse model	*A. muciniphila* (5 × 10^9^ CFU/200 µl) 6 months	•Improve glucose tolerance •Improve intestinal mucosal barrier damage •Ameliorate memory impairment and maintain attention. •↓Aβ plaque deposition and Aβ levels	(Ou et al., 2020)
	Conventionally-raised transgenic APPPS1 mouse model	16S rRNA gene V3-V4 regions sequencing	•↓*A. muciniphila*, *Allobaculum* •↑*Rikenellaceae* •*A. muciniphila* is related to Aβ	(Harach et al., 2017)
**PD**	38 PD patients 34 healthy controls	16S rRNA gene V4 regions sequencing	•Different microbiota diversity and composition between sigmoid mucosal biopsies and fecal samples •*↑Blautia*, *Coprococcus*, and *Roseburia* in PD fecal microbiota •*↓Faecalibacterium* in mucosal microbiota •*↑Ralstonia* in mucosal microbiota	(Keshavarzian et al., 2015)
	197 PD patients 103 healthy controls	16S rRNA gene V4 regions sequencing	•*↑Bifidobacterium*, *Collinsella*, *Bilophila*, and *Akkermansia* in PD •↓*Roseburia*, unclassified *Lachnospiraceae* genus, *Faecalibacterium* •*↓*carbohydrate fermentation and butyrate synthesis capacity •↑proteolytic fermentation and production of deleterious amino acid metabolites	(Cirstea et al., 2020)
	31 Early-stage L-DOPA-naïve PD patients 28 healthy controls	Shotgun metagenomic sequencing	•*↑A. muciniphila*, *Alistipes shahii* and unclassified Firmicutes •↓*Prevotella copri*, *Eubacterium biforme*, and *Clostridium saccharolyticum* •Altered β-glucuronate and tryptophan metabolism	(Bedarf et al., 2017)
	147 typical PD patients 162 controls	16S rRNA gene V3-V4 regions sequencing	•↑Species richness in PD •↓Pielou index in female PD patients •Bray-Curtis dissimilarities different •Genus level: ↑*Akkermansia*, *Christensenella*, *Anaerotruncus*, *Lactobacillus*, *Streptococcus*, *Bilophila*, and *Acidaminococcu*s; ↓*Turicibacter* •Species level: *↑A. muciniphila*, *Christensenella minuta*, *Anaerotruncus colihominis*, *Ruminococcus bromii*, *Ruminococcus torques*, and *Roseburia intestinalis*; ↓*Turicibacter sanguinis* •*Lactobacillus* positively correlated with the disease duration	(Baldini et al., 2020)
	27 hospitalized PD patients 44 healthy subjects	16S rRNA gene V3-V4 regions sequencing	•Different α- and β-diversity •↑*A. muciniphila*, *Eubacterium biforme*, and *Parabacteroides merdae* •↓*Faecalibacterium prausnitzii*, *Ruminococcus albus*, and *Blautia faecis*	(Zapała et al., 2021)
	Rotenone-induced PD mouse model	unpredictable restraint stress for 12 weeks, rotenone (10 mg/kg/day) orally in last 6 weeks	•Intestinal hyper-permeability •↓ZO-1, Occludin, Claudin1 •↑N-tyrosine •↑inflammation in glial cells •Endotoxemia •↓resting microglia;↑ dystrophic/phagocytic microglia •↑*A. muciniphila*; ↓*Coriobacteriaceae* •*A. muciniphila* were positively correlated with serum LPS	(Dodiya et al., 2020)
	MPTP-induced PD mouse model	MPTP (30 mg/kg) 5days +KRG (100 mg/kg) 12 days	•↓dopaminergic neuronal death •↓activation of microglia and astrocytes •↓accumulation of α-synuclein in the SN •Inflammation-related: ↓*A. muciniphila*, *Ruminococcus albus* •Anti-inflammatory related: ↑*Eubacterium, Flavonifractor, Mucispirillum*	(Jeon et al., 2021)
**MS**	40 healthy controls 199 RRMS patients 44 progressive patients EAE mouse model with *Akkermansia* strains	16S rRNA gene V4 region sequencing	•↑Shannon diversity, and richness in MS •Altered β-diversity in MS •Progressive MS: ↑Enterobacteriaceae, Ruminococcaceae FJ366134 and Clostridaceae g24 FCEY; ↓*Dorea longicatena*, *Anaerococcus vaginalis*, and *Blautia faecis* •RRMS and Progressive MS: ↑*Akkermansia*, *Clostridium bolteae*;↓ *Dorea formicigenerans*, unclassified *Blautia* •*Akkermansia* was negatively correlated with EDSS and MRI burden of MS •*Akkermansia* strain BWH-H3 ameliorated EAE *via* reducing RORγt^+^ and IL-17-producing γδ T cells	(Cox et al., 2021)
	62 RRMS, 15SPMS, 21 atypical MS 20 NMOSD 55 healthy controls	16S rRNA gene V1-V2 regions and whole metagenomic sequencing	•No changed α-diversity; altered β-diversity among groups •Compared with HC: •↑*Bifidobacterium* and ↓*Megamona*s in RRMS; ↑*Streptococcus* in RRMS and SPMS; ↓*Roseburia* in SPMS; ↑*Alistipes* in NMOSD •↑*A. muciniphila*, *Clostridium leptum*, *Streptococcus parasanguinis*, *Streptococcus salivarius/thermophilus* in RRMS; ↓*Eubacterium rectale*, *Ruminococcus* sp. 5_1_39BFAA, and *Megamonas funiformis* in RRMS •↓fecal acetate, propionate, and butyrate in RRMS	(Takewaki et al., 2020)
	71 untreated MS patients 71 healthy controls	16S rRNA gene V4 region sequencing	•No major shifts in microbial community structure •↑*A. muciniphila*, and *Acinetobacter calcoaceticus*; ↓*Parabacteroides distasoni* •*A. muciniphila* stimulate TH1 differentiation, build proinflammatory environment.	(Cekanaviciute et al., 2017)
	34 monozygotic twin pairs discordant for MS	16S rRNA gene V3-V5 regions sequencing and metagenomic sequencing	•No overt differences in alpha or beta diversity •↑*A. muciniphila* in untreated MS twin siblings	(Berer et al., 2017)
	EAE mouse model	THC+CBD	•↓inflammatory cytokines: IL-17 and IFN-γ •↑anti-inflammatory cytokines: IL-10 and TGF-β •↓A. muciniphila •↓LPS biosynthesis •↑butyric, isovaleric, and valeric acids	(Al-Ghezi et al., 2019)
**Stroke**	16 healthy volunteers; 10 LI patients; 10 AI patients; 10 PI patients	16S rRNA gene V3 region sequencing	•Altered bacterial diversity in stoke patients •↑Cyanobacteria and Fusobacteria in LI patients •↑Actinobacteria in AI patients •↑Verrucomicrobia, Synergistetes, and Proteobacteria in PI patients •*Akkermansia* is the predominant genera in PI patients •*Pseudomonas*, *Sphingomonadaceae*, and *Akkermansia* as markers for PI patients	(Xiang et al., 2020)
	30 CI patients; 30 healthy controls	16S rRNA gene V1-V2 regions sequencing	•Unaltered bacterial diversity in CI patients •↑*Odoribacter*; *Akkermansia*; *Ruminococcaceae*; *Victivallis* in CI	(Li et al., 2019)
	8 cerebral infarction patients; 2 ischemia patients; 10 healthy volunteers	16S rRNA gene V4 region sequencing	•↑*Escherichia*, *Megamonas*, *Dialister*, *Bifidobacterium* and *Ruminococcus* in patients •↓*Bacteroides*, *Parabacteroides*, *Akkermansia*, *Prevotella* and *Faecalibacterium* in cerebral infarction patients than controls •*Escherichia*, *Dialister* and *Bifidobacterium* in ischemia patients than controls	(Ji et al., 2017)
	232 acute ischemic stroke patients: 88 PSD 144 non-PSD	16S rRNA gene V3-V4 regions sequencing	•*↑Streptococcus*, *Akkermansia*, and *Barnesiella* in PSD patients •↓*Escherichia-Shigella, Butyricicoccus*, and *Holdemanella* in PSD patient •*Akkermansia, Barnesiella*, and *Pyramidobacter* were positively correlated with HAMD-17 score	(Yao et al., 2023)
	Focal cerebral ischemia mouse model	16S rRNA gene V3-V4 regions sequencing	•**↑** *Akkermansia*, *Parabacteroides*, *Anaerotruncus*, *Alistipes*, *Bacteroides* in post-stroke mice •**↑** *A. muciniphila*, *Parabacteroides goldsteinii, Anaerotruncus colihominis, Alistipes shahii* and *Roseburia intestinalis* in post-stroke mice •*Akkermansia* was positively correlated with *Ruminococcus*, *Alistipes*, *Bacteroides* and *Parabacteroides*, but negatively correlated with *Staphylococcus* and *Streptococcus*	(Stanley et al., 2018)
**ASD**	23 ASD children; 22 SIB children; 9 controls	Quantitative real-time PCR	•↓*A. muciniphila*, *Bifidobacterium* spp.	(Wang et al., 2011)
	48 ASD children; 48 healthy children	16S rRNA gene V3-V4 regions sequencing	•↑Bacteroidetes/Firmicutes in ASD •↓*A. muciniphila*, *Dialister invisus*, *Escherichia coli*, *Bacteroides fragilis*, *Haemophilus parainflfluenzae*, *Flavonifractor plautii* in ASD •↑*Bacteroides coprocola*, *Bacteroides vulgatus*, *Eubacterium eligens*, *Prevotella copri*, *Roseburia faecis* in ASD •metabolic disruptions in ASD	(Zou et al., 2020)
	20 neurotypical children 20 autistic children	16S rRNA gene V2-V3 regions sequencing	•Autism children *VS* neurotypical children: ( ↑*Akkermansia*, *Ruminococcus* ( ↓*Prevotella*, *Coprococcus*, *Blautia*, *Collinsella*	(Kang et al., 2013)
	BTBR^T+tf/j^ (BTBR) mouse model	ketogenic diet for 10-14 days	•triggers gut microbiota remodeling •↑*A. muciniphila* in the cecum and feces of BTBR animals	(Newell et al., 2016)
	*Fmr1* KO mouse model	FMT for the latter 4 weeks	•FMT attenuates cognitive deficits in Fmr1 KO mice •FMT ameliorates social novelty preference •↓*A. muciniphila* in *Fmr1* KO mice •*A. muciniphila* reduces TNF-α in the cortex and hippocampus •FMT normalized the increased expression level of Iba1	(Goo et al., 2020)
	VPA induced mouse model	*A. muciniphila* (1 × 10^9^ CFU/ml) 30μl for 14 days	•*A. muciniphila* alleviates the social deficit in the VPA-induced mouse model •*A. muciniphila* enhanced activation of VTA dopaminergic neurons during social interaction. •Early-life *A. muciniphila* supplementation induced a wide range of metabolic alterations	(Liu X. et al., 2022)

5-HT, 5-hydroxytryptamine; AD, Alzheimer’s disease; AI, non-lacunar acute ischemic infarction; ASD, autism spectrum disorder; BDNF, brain-derived neurotrophic factor; CBD, cannabidiol; CFU, colony forming unit; CI, cerebral ischemic stroke; CRS, Chronic restraint stress; EAE, Experimental autoimmune encephalomyelitis; EDSS, expanded disability status score; FMT, fecal microbiota transplantation; HAMD-17, 17-Hamilton Depression Rating Scale; HC, healthy controls; IFN, interferon; IL, interleukin; IS, ischemia; KO, knock out; KRG, Korean red ginseng; LI, lacunar infarction; LPS, lipopolysaccharide; MMSE, Mini-mental State Examination; MPTP, 1-methyl-4-phenyl-1,2,3,6-tetrahydropyridine; MRI, magnetic resonance imaging; MS, multiple sclerosis; NMOSD, neuromyelitis optica spectrum disorder; PD, Parkinson’s disease; PI, post-ischemic stroke; PSD, post-stroke depression; RRMS, Relapsing remitting multiple sclerosis; SIB, siblings; SN, Substantia Nigra; SPMS, secondary progressive multiple sclerosis; TGF, transforming growth factor; THC, delta-9-tetrahydrocannabinol; TNF, tumor necrosis factor; VPA, valproic acid; VTA, ventral tegmental area; WAIS, Wechsler Adult Intelligent Scale.

### 3.1 Depression

Depression is one of the most common mental health disorders worldwide. Individuals affected by depression experience symptoms such as persistent low mood, loss of interest, difficulty concentrating, psychomotor retardation or agitation, insomnia and other sleep disturbances, or suicidal thoughts. The World Health Organization estimates that approximately 350 million people suffer from depression, and the condition may result in disabilities or mortality (Smith, 2014). Owing to the peculiarities of depression and the subjective nature of its diagnosis, the number of undiagnosed patients with subclinical depressive symptoms is estimated to be even higher. At present, the etiology of depression is not clear, although biochemical alterations in monoamines and their receptors are suspected to contribute to the development of depression (Ruiz et al., 2018). Experimental and genetic research has revealed several mechanisms for the development of depression, including dysregulation of the hypothalamus-pituitary-adrenal disorder (HPA) axis in response to stress, inflammation, impaired neural plasticity, disrupted neural circuits, and dysfunction of nerve regulation systems such as the monoamine and endocannabinoid (eCB) systems (Chevalier et al., 2020; Mlynarska et al., 2022).

Recent studies have revealed that the gut microbiota plays an important role in the pathogenesis of depression (Jiang et al., 2015; Liang et al., 2018; Ling Z. et al., 2022). Over the course of evolution, the gut microbiome, a virtual endocrine organ that communicates with the CNS via the MGBA, has developed a bidirectional communication relationship with its host (Clarke et al., 2014; O’mahony et al., 2015). *A. muciniphila* is believed to participate in this pathway by producing metabolites that influence neuroendocrine signaling in the brain. Furthermore, studies have observed decreased levels of *A. muciniphila* in mice exhibiting depression-like behavior following social defeat (Mcgaughey et al., 2019) and rats displaying depression-like behavior after chronic paradoxical sleep deprivation (Park et al., 2020). Studies have shown that individuals with depression exhibit reduced levels of BDNF in the brain (Takebayashi et al., 2012; Jiang et al., 2015). The neurotrophic hypothesis proposes that a decrease in BDNF is implicated in the pathophysiology of depression, while an increase in BDNF is essential for the therapeutic effect of antidepressants (Yohn et al., 2017). BDNF is a neurotrophic factor, which is widely distributed in the hippocampus and closely related to neuronal regeneration and repair (Rakhit et al., 2005). A decrease in BDNF mRNA levels is observed in the hippocampus of chronically stressed rats and patients with depression (Duman and Monteggia, 2006). However, *A. muciniphila* increases the expression of BDNF mRNA in the hippocampus, indicating its potential to enhance synaptic signaling pathways, promote neuronal connectivity, and improve the condition of patients with depression (Ding et al., 2021). Clinically, patients with depression exhibit reduced levels of 5-HT, which is a protective factor (Tundo et al., 2015). According to the monoamine deficiency hypothesis, depression is caused by a deficiency in monoamine transmitters, such as 5-HT, within the brain (Gordon and Goelman, 2016). *A. muciniphila* directly influences the host 5-HT system and can increases 5-HT levels in the host intestine (Cheng R. et al., 2021). As previously mentioned, Amuc_1100 upregulates the expression of 5-HT in the intestine (Wang et al., 2021).

Ding et al. found that oral supplementation with *A. muciniphila* improves depression by modulating cholinergic synapses, fat digestion and absorption, aromatic compound degradation, vitamin digestion and absorption, fatty acid degradation, butanoate metabolism, carbon metabolism, biosynthesis of pantothenic acid and CoA, metabolic pathways, and digestion and absorption (Ding et al., 2021). *A. muciniphila* also increased the expression of two metabolites, edaravone and β-alanyl-3-methyl-L-histidine, both of which showed a tendency to restore BDNF expression in the hippocampus. Furthermore, both metabolites significantly reduced corticosterone concentrations. In addition, edaravone significantly increased serotonin concentration, while β-alanyl-3-methyl-l-histidine increased dopamine concentration. The experimental results also showed that *A. muciniphila* alleviated as the symptoms of depression by influencing the levels of monoamine neurotransmitters and BDNF. *A. muciniphila* increases the expression of BDNF mRNA in the hippocampus, indicating that *A. muciniphila* can enhance synaptic signaling pathways and neuronal connections (Ding et al., 2021). Recently, Sun et al. found that *A. muciniphila* and Amuc_1100 could alleviate anxiety- and depression-like behaviors in mice (Sun et al., 2023). Following treatment with *A. muciniphila* and Amuc_1100, levels of BDNF and its receptor, TrkB, were restored in the hippocampus and cortex. TrkB undergoes autophosphorylation after interacting with BDNF, which participates in signaling cascades that regulate neuronal migration and differentiation (Andreska et al., 2020). Previous studies have also demonstrated the association of both BDNF and TrkB with mood disorders and antidepressant effects (Castrén and Kojima, 2017; Hing et al., 2018). Therefore, *A. muciniphila* and Amuc_1100 may alleviate antibiosis-induced anxiety and depression by regulating the BDNF/TrkB signaling pathway, or by increasing 5-HT levels in the serum and hippocampus (Sun et al., 2023). Additionally, *A. muciniphila* may assist in alleviating the symptoms of depression by regulating gut inflammation. Studies have shown that individuals with depression have higher levels of inflammation, and that *A. muciniphila* may help mitigate this inflammation by producing anti-inflammatory molecules. In conclusion, *A. muciniphila* may play a critical role in mood regulation and depression prevention by affecting the MGBA, regulating inflammation, producing SCFAs, and modulating BDNF levels.

### 3.2 Alzheimer’s disease

AD is characterized by memory impairment, loss of visual skills, performance decline, and behavioral changes (Kuhlmann et al., 2017). Currently, AD is regarded as the main cause of dementia and is quickly becoming one of the most expensive, lethal, and burdensome diseases of this century (Scheltens et al., 2021). The neuropathology of AD is characterized by the accumulation and aggregation of amyloid-β (Aβ) and tau proteins in the brain, causing excessive phosphorylation of tau proteins, leading to neurofibrillary tangles, neuroinflammation, and neuronal and synaptic loss (Griciuc and Tanzi, 2021; Knopman et al., 2021). Aβ is a peptide produced by the proteolytic hydrolysis of APP by β-and γ-secretase. Aβ40 and Aβ42 are highly toxic and are highly likely to aggregate and exert neurotoxic effects. Hence, they are widely recognized as a key biomarker for AD (Kuhlmann et al., 2017). Accumulated Aβ also increases intestinal permeability, promoting the expression of proinflammatory cytokines such as IL-17A and IL-22, which can cross the gut mucosal barrier and BBB and trigger neuroinflammation. Studies have shown a bidirectional relationship between metabolic syndromes such as obesity or insulin resistance and AD. On one hand, individuals with metabolic syndromes have a higher risk of cognitive impairment and AD (Craft, 2005; De Felice, 2013). On the other hand, the presence of AD pathology, such as Aβ deposits, can interfere with insulin signaling and contribute to insulin resistance (Carvalho et al., 2013; Barbagallo and Dominguez, 2014). These findings suggest that insulin resistance is a key link between metabolic dysfunction and AD.

In AD mouse models, a significant decrease in the number of *A. muciniphila* is observed (Harach et al., 2017). A reduced amount of *A. muciniphila* is associated with impairment of the intestinal barrier (Grander et al., 2018), which is linked to cognitive function deficits and learning and memory impairment in AD mouse models (Ou et al., 2020). Increased intestinal permeability can lead to the translocation of bacteria-derived LPS and amyloid proteins from the gut into the bloodstream and eventually the brain, which further aggravates intestinal permeability (Pistollato et al., 2016). The damaged intestinal barrier can be repaired by treatment with *A. muciniphila*, which reduces blood endotoxin levels and improves insulin resistance (Everard et al., 2013; Shin et al., 2014). Ou et al. discovered that *A. muciniphila* may increase the number of goblet cells and promote mucus secretion to repair the damaged gut mucosal barrier, thereby reducing LPS and other proinflammatory substances. This repair mechanism improves metabolic levels, alleviates insulin resistance, reduces Aβ protein deposition, and protects nerves from damage (Ou et al., 2020). In addition, daily administration of the Chinese strain *A. muciniphila* GP01 to APP/PS1 mice for 6 months not only impacted the integrity of the gut mucosal barrier (Ou et al., 2020), but also resulted in decreased Aβ deposition and led to improved performance in the Y-maze test. *A. muciniphila* intervention can reduce Aβ40-42 in the cerebral cortex of AD animal models and alleviate cognitive disorders, such as spatial learning and memory deficits (Ou et al., 2020). These findings highlight the potential of *A. muciniphila* treatment in improving gut mucosal barrier dysfunction, as well as glucose and lipid metabolism disorders associated with cognitive impairment. However, it should be noted that the patterns observed in humans regarding the effects of *A. muciniphila* have been inconsistent. Increases in the abundance of the gut microbiota in patients with AD have been widely reported (Zhuang et al., 2018; Ling et al., 2020b; Khedr et al., 2022; Wang Y. et al., 2022). This challenged the positive role of *A. muciniphila* in AD animal models. However, a modified Mediterranean-ketogenic diet, along with increased *A. muciniphila*, has been shown to ameliorate markers of AD in patients with mild cognitive impairment (Nagpal et al., 2019). These conflicting results between human and animal studies stresses the need for further research to fully comprehend the roles and mechanisms of *A. muciniphila* in the development and progression of AD prior to considering it as a therapeutic target.

### 3.3 Parkinson’s disease

PD is a neurodegenerative disease characterized by motor symptoms such as stiffness, resting tremors, bradykinesia, postural instability, and gait disturbances, in addition to non-motor symptoms such as cognitive, neuropsychiatric, and autonomic impairment (Reich and Savitt, 2019). With the aging global population, global burden of disease studies project that the number of cases of PD will reach approximately 13 million by 2040 (Collaborators, G.B.D.P.S.D., 2018). The exact etiology of PD has not yet been clarified; however, studies have shown that the loss of dopaminergic activity, presence of Lewy bodies, intestinal exposure to neurotoxins, gut dysbiosis, cytokine-induced toxicity, oxidative damage from inflammation, and aging are all associated with the pathogenesis and progression of PD (Barichella et al., 2016). Dopaminergic neurons in the substantia nigra pars compacta (SNpc) appear to be particularly vulnerable to aggregates of aSyn, which can activate immune cells, including microglia in the brain (Kim C. et al., 2013). The abnormally soluble oligomeric conformation of aSyn, known as protofibrils, is widely recognized as a toxic agent that disrupts cellular homeostasis and contributes to neuronal death by influencing various intracellular processes (Stefanis, 2012). Elevated levels of glycoprotein non-metastatic melanoma protein B (GPNMB) have been observed in the blood of patients with PD and its expression is correlated with disease severity, suggesting that GPNMB may serve as a biomarker for PD progression (Smith and Schapira, 2022). GPNMB is a transmembrane glycoprotein (Mollenhauer and Caf., 2022), and genetic variations in the form of single nucleotide polymorphisms (SNPs) in this gene are associated with an increased risk of PD (Kia et al., 2021).

Similar to the findings in AD, gut dysbiosis has been observed even in the prodromal phase of PD, with enrichment of *A. muciniphila* occurring in patients with PD (Keshavarzian et al., 2015; Bedarf et al., 2017; Hill-Burns et al., 2017; Baldini et al., 2020; Cirstea et al., 2020; Zapała et al., 2021). Additionally, an increased number of *A. muciniphila* has been found in MPTP-induced (Jeon et al., 2021) and rotenone-treated PD mouse models (Dodiya et al., 2020). These findings suggest that *A. muciniphila* plays an important role in the pathogenesis of PD, and its increase may serve as a potential early biomarker for PD. The positive correlation between *A. muciniphila* and PD neural pathologies may be attributed to its role in mucin degradation, which can aggravate gut inflammation and permeability, leading to elevated endotoxemia and systemic inflammation. Notably, mucus degradation by *A. muciniphila* may result in a compensatory increase in mucus synthesis and exert anti-inflammatory effects in the host (Derrien et al., 2017). Using metagenomic analysis, Qian et al. found that *A. muciniphila*-related proteins might play an important role in the progression of PD (Qian et al., 2020). In addition, Ansaldo et al. found that the specific T cell responses induced by *A. muciniphila* can vary depending on other species present in the gut microbiota (Ansaldo et al., 2019). This variability may help explain why *A. muciniphila*, generally believed to mediate anti-inflammatory effects, is found in excessive amounts in individuals with PD. However, it remains unknown whether *A. muciniphila* can alleviate cognitive decline in patients with PD and dementia. Further investigation is warranted to fully understand the roles and mechanisms of *A. muciniphila* in PD, especially considering that it is a potential probiotic treatment for PD.

### 3.4 Multiple sclerosis

MS is a common, chronic, nontraumatic, and disabling autoimmune disease that primarily affects the CNS by targeting the myelin sheath. It is characterized by demyelination, loss of oligodendrocytes, reactive glial cell proliferation, and axonal degeneration (Ransohoff et al., 2015). The disease gradually erodes the CNS through the immune system, leading to a decline in the physical flexibility of patients (Pettigrew et al., 2016); hence, progressive MS is associated with increased physical disability and a significant impairment of the quality of life, including symptoms such as depression and fatigue (Cox et al., 2021). MS typically manifests in young individuals aged 20–40 years, and its occurrence and prevalence are increasing globally (Oh et al., 2018). Although the exact pathogenesis of MS is unknown, it is believed to involve a complex interplay between genetic and environmental factors. Among the environmental factors, the gut microbiota has been found to influence both the susceptibility to MS and the progression of the disease. The gut microbiota has the ability to modulate the differentiation and function of peripheral immune cells that regulate CNS inflammation (Cheng Y. et al., 2021); thus, studies have highlighted their significant role in autoimmune CNS disorders (Berer et al., 2011; Lee et al., 2011).

Gut dysbiosis has been observed in various stages of MS, suggesting an active involvement of the gut microbiota in the development of the disease (Takewaki et al., 2020). Among gut bacteria that are altered in individuals with MS, *A. muciniphila* has attracted considerable attention. Using 16S ribosomal RNA gene sequencing, Baranzini et al. observed that patients with MS had higher levels of *A. muciniphila* and lower levels of SCFA-producing bacteria in their guts compared to those in healthy individuals (Cekanaviciute et al., 2017), which is consistent with the findings of other studies (Berer et al., 2017; Tankou et al., 2018; Al-Ghezi et al., 2019; Kozhieva et al., 2019; Takewaki et al., 2020). *A. muciniphila* can induce proinflammatory responses in human peripheral blood mononuclear cells and in monocolonized mice, which may contribute to the development of autoimmune diseases like MS (Cekanaviciute et al., 2017). Cekanaviciute et al. found a significant positive correlation between the relative abundance of *Akkermansia* and the differentiation of IFN-γ^+^ Th1 lymphocytes (Cekanaviciute et al., 2018), further suggesting the involvement of *A. muciniphila* in MS pathogenesis. In contrast to the notion that a higher relative abundance of *A. muciniphila* in MS is detrimental, Cox et al. identified a negative association between the abundance of *A. muciniphila* and disability, and a positive correlation between *A. muciniphila* and brain volume, demonstrating that *A. muciniphila* could also play a beneficial role. Consistent with this finding, their study revealed that *Akkermansia* isolated from MS patients was able to ameliorate experimental autoimmune encephalomyelitis (EAE) by reducing RORγt^+^ and IL-17-producing γδ T cells (Cox et al., 2021). This suggests an overall beneficial role of *A. muciniphila*, similar to that observed in obesity, diabetes, and age-related complications (Gurung et al., 2020). Convincing evidence identified IL-17-producing γδ T cells as the initiators of the inflammatory process in EAE (Mcginley et al., 2018). Additionally, Colpitts et al. found that mice with higher levels of *A. muciniphila* exhibited slower disease progression in a non-obese diabetic progressive EAE model (Colpitts et al., 2017). Koutrolos et al. observed that *A. muciniphila* can up-regulate TGF-β and down-regulate IL-6 and IL-1β in mesenteric lymph node dendritic cells, which is beneficial to control the Treg expansion of effector T cells during EAE (Koutrolos et al., 2014). Liu et al. demonstrated that oral administration of miR-30d, found in the feces of MS patients, increased the number of Tregs and suppressed EAE symptoms in mice by increasing *A. muciniphila* in the gut (Liu et al., 2019). These findings suggest that elevated levels of *A. muciniphila* in MS patients may be a compensatory beneficial response of the MS microbiota. However, *A. muciniphila* also exerts strain-specific physiological functions, as inter-species or inter-genetic differences of *A. muciniphila* have been observed (Liu et al., 2021), implying that not all strains of *A. muciniphila* are beneficial to the host. Different strains of the same species may exhibit contradictory effects on the same disease, suggesting the importance of analyzing bacterial functions at strain level (Wu G. et al., 2017). Thus, the beneficial effects of *A. muciniphila* may be strain-specific, warranting further studies on the effect of different strains of *A. muciniphila* on MS.

### 3.5 Strokes

A stroke can be classified as ischemic or hemorrhagic based on neuropathology. Ischemic strokes account for 85% of all stroke cases, while hemorrhagic strokes account for approximately 15% (Parr et al., 2017). A stroke is caused by the interruption of blood supply to regions of the brain, which can lead to permanent neurological deficits or death (Hossmann, 2006). It is a global health problem and is now the second leading cause of death and the third leading cause of disability (Parr et al., 2017). Brain damage caused by ischemic stroke is the result of a complex series of neuropathophysiological events, including oxidative stress, neuroinflammation, apoptosis, excitotoxicity, amyloid production, and tau dysfunction (Pluta et al., 2020; Radenovic et al., 2020; Ulamek-Koziol et al., 2020). Studies revealed that bacterial pneumonia is the main cause of death after a stroke (Chamorro and Urra, 2007). Selective bacterial translocation from the host gut microbiota to the lungs is the key factor causing bacterial pneumonia, suggesting that the gut epithelial mucosa and gut microbiota play a significant role in post-stroke mortality (Stanley et al., 2018).

Gut dysbiosis has been observed in both rodents and humans following the onset of an acute ischemic stroke. Singh et al. found that a stroke can induce gut dysbiosis and that changes in the gut microbiota can affect neuroinflammatory and functional outcomes after brain injury, while fecal microbiota transplantation (FMT) can improve stroke outcomes by normalizing post-stroke dysbiosis (Singh et al., 2016). Benakis et al. observed that antibiotic treatment-induced gut dysbiosis influences post-stroke neuroinflammation and outcome (Benakis et al., 2016). Clinical studies have reported a significant increase in *A. muciniphila* in the feces of patients who have experienced cerebral ischemic strokes (Li et al., 2019; Tan et al., 2021; Yao et al., 2023), proposing it as a gut microbiological marker following ischemic strokes (Xiang et al., 2020). In a rodent stroke model, an increase in *A. municiphila* was observed compared to that in control animals (Stanley et al., 2018; Ryuk et al., 2022). Patterns of changes in the abundance of *A. muciniphila* in stroke patients are not always consistent, similar to the observations in patients with AD and PD. Contrary to the results described above, a decrease in *A. muciniphila* was observed in patients with post-stroke (Ji et al., 2017). The role of *A. muciniphila* in stroke patients remains unclear. Stanley et al. demonstrated that *A. muciniphila* may prevent the migration of pathogens to epithelial cells through active inhibition, resulting in reduced translocation and dissemination of pathogens in the lungs after a stroke (Stanley et al., 2016). *A. muciniphila* can also promote wound healing and strengthen epithelial integrity to repair the intestinal mucosa. Some researchers propose that *A. muciniphila* can induce mucus production and Reg3γ expression in the colon, remodeling the gut microbiota (Hänninen et al., 2018). In addition, *A. muciniphila* may inhibit the production of TNF-α and free radicals by interacting with TLRs in intestinal epithelial cells, which is associated with the reduction of serum TNF-α, IL-6, and IL-10 levels, positively impacting post-stroke recovery (Akhoundzadeh et al., 2018; Zhong et al., 2021). Further studies are required to determine the role of *A. muciniphila* in strokes.

### 3.6 Autism spectrum disorder

ASD is a complex neurodevelopmental disorder characterized by persistent deficits in social communication and interactions, with limited interests and repetitive behaviors (Persico et al., 2019). ASD typically emerges in early childhood and its prevalence is estimated at 1–2% based on numerous studies conducted in Asia, Europe, and North America (Valentino et al., 2021). Research and intervention in ASDs have become a pressing priority for public health due to the associated healthcare costs and complexity of the disorder. Multiple mechanisms such as decreased neurogenesis, an imbalance of excitatory/inhibitory neurotransmission, and dysregulation of the immune response, have been proposed as potential contributors to the onset of ASD (Adhya et al., 2021; Baranova et al., 2021; Cast et al., 2021). A recent study has provided direct and strong evidence suggesting that the gut microbiota plays a role in mediating the core symptom of ASD, the social behavioral phenotype (Buffington et al., 2021). While the exact etiology and pathology of ASD are still unclear, MGBA dysfunction is emerging as a prominent factor contributing to the development of autistic behaviors.

Increasing evidence supports the involvement of the gut microbiota in ASD (Foster, 2022). Altered gut microbiota composition and metabolic activities have also been detected in both many previous clinical (Finegold et al., 2010; Gondalia et al., 2012; De Angelis et al., 2013; Kang et al., 2013; Kushak et al., 2017; Strati et al., 2017; Coretti et al., 2018; Wang et al., 2019) and pre-clinical studies (Hsiao et al., 2013; De Theije et al., 2014; Buffington et al., 2016; Coretti et al., 2017; Kim et al., 2017; Liu et al., 2018; Sauer et al., 2019). Conflicting results have been reported for the involvement of *A. muciniphila* in ASD. Two sequencing studies have found increased levels of *A. muciniphila* in patients with ASD (De Angelis et al., 2013; Kang et al., 2013), while other studies have reported reduced levels of *A. muciniphila* in the gut microbiota of children with ASD (Finegold et al., 2010; Wang et al., 2011; Kang et al., 2013; Zou et al., 2020). Consistent with a previous study, the decreased number of *A*. *muciniphila* suggests abnormally elevated gut permeability in children (De Magistris et al., 2010). In a murine model of ASD, Newell et al. observed that a ketogenic diet could elevate the levels of *A. muciniphila* in cecal and fecal matter. This increase is associated with the alleviation of some of the neurological symptoms related to ASD (Newell et al., 2016). Using an *Fmr1* knock out (KO) ASD mouse model, Goo et al. found a significant decrease in the number of *A. muciniphila*. However, FMT with feces from wild-type mice normalized the levels of *A. muciniphila*, reduced the expression level of TNF-α, and inhibited the activation of microglia cells (Goo et al., 2020). reestablishing the gut microbiota ameliorated autistic-like behaviors, especially cognitive dysfunction and deficits in social novelty preference behaviors. The beneficial effects of FMT on ASD involving the regulation of *A. muciniphila*, suggest that *A. muciniphila* might play a key role in ASD development. However, whether the decrease in *A. muciniphila* is an important causative factor of the autistic-like behavior observed in *Fmr1* KO mice remains uncertain. Further research is required to validate this hypothesis. Recently, Liu et al. discovered that supplementation with *A. muciniphila* during early life could mitigate social deficits induced by exposure to valproic acid through activating dopaminergic neurons in the ventral tegmental area (Liu X. et al., 2022). These findings suggest that *A. muciniphila* may serve as a potential probiotic treatment for ASD, although the exact mechanism by which it influences ASD requires further investigation.

## 4 Conclusions

*A. muciniphila* has recently emerged as a promising potential probiotic with significant metabolic and immunological properties. It influences brain and behavioral functions through the MGBA. Mounting clinical and preclinical evidence suggest that differences in the number of *A. muciniphila* play an active role in the development of various neuropsychiatric disorders, such as depression, anxiety, AD, PD, MS, stroke, and ASD. Its effects are mediated through the production of neuroactive metabolites, adjustment of the gut microbiota, modulation of the gut mucosal barrier, and regulation of host immunity and metabolism. *A. muciniphila* is considered an important diagnostic and therapeutic target for these neuropsychiatric disorders, and could be regarded as a “friend” in terms of host microbiota. However, contradictory findings have been reported regarding the relationship between the relative abundance of *A. muciniphila* and several neuropsychiatric disorders, making it challenging to determine the precise role of *A. muciniphila* in some of these disorders. However, there is still insufficient evidence to classify *A. muciniphila* as a “foe” based on current studies. Nevertheless, there is still a long way to go to develop *A. muciniphila* as a probiotic for neuropsychiatric disorders. Most studies have focused on correlation analysis between the number of *A. muciniphila* and neuropsychiatric disorders, but little is known about the causal relationship. Further research utilizing germ-free neuropsychiatric disorder animal models, organoid models, specific strains of *A. muciniphila*, and comprehensive behavioral and immunological analyses is necessary to provide more mechanistic insights to support the use of *A. muciniphila* for the prevention and treatment of neuropsychiatric disorders, prior to evaluating its efficacy in human clinical trials.

## Author contributions

Conceptualization, ZL and WH. Methodology, WL, YC, JG, XL, LS, QK, NZ, ZL, and WH. Writing-original draft preparation, WL, YC, ZL, and WH. Critical revision and editing, XL, LS, QK, and ZL. Review and editing, ZL and WH. All authors have read and agreed to the published version of the manuscript.

## References

[B1] Adhya D. Swarup V. Nagy R. Dutan L. Shum C. Valencia-Alarcon E. P.. (2021). Atypical neurogenesis in induced pluripotent stem cells from autistic individuals. Biol. Psychiatry 89 (5), 486–496. doi: 10.1016/j.biopsych.2020.06.014 32826066 PMC7843956

[B2] Akhoundzadeh K. Vakili A. Shadnoush M. Sadeghzadeh J. (2018). Effects of the oral ingestion of probiotics on brain damage in a transient model of focal cerebral ische mia in mice. Iranian J. Med. Sci. 43 (1), 32–40. PMC5775992 29398750

[B3] Al-Ghezi Z. Z. Busbee P. B. Alghetaa H. Nagarkatti P. S. Nagarkatti M. (2019). Combination of cannabinoids, delta-9-tetrahydrocannabinol (THC) and cannabidiol (CBD), mitigates experimental autoimmune encephalomyelitis (EAE) by altering the gut microbiome. Brain Behav. Immun. 82, 25–35. doi: 10.1016/j.bbi.2019.07.028 31356922 PMC6866665

[B4] Alhawi M. Stewart J. Erridge C. Patrick S. Poxton I. R. (2009). Bacteroides fragilis signals through toll-like receptor (TLR) 2 and not through TLR4. J. Med. Microbiol. 58 (Pt 8), 1015–1022. doi: 10.1099/jmm.0.009936-0 19528164

[B5] Ambort D. Johansson M. E. Gustafsson J. K. Nilsson H. E. Ermund A. Johansson B. R.. (2012). Calcium and pH-dependent packing and release of the gel-forming MUC2 mucin. Proc. Natl. Acad. Sci. U.S.A. 109 (15), 5645–5650. doi: 10.1073/pnas.1120269109 22451922 PMC3326483

[B6] Andreska T. Luningschror P. Sendtner M. (2020). Regulation of TrkB cell surface expression-a mechanism for modulation of neuronal responsiveness to brain-derived neurotrophic factor. Cell Tissue Res. 382 (1), 5–14. doi: 10.1007/s00441-020-03224-7 32556728 PMC7529634

[B7] Anhe F. F. Marette A. (2017). A microbial protein that alleviates metabolic syndrome. Nat. Med. 23 (1), 11–12. doi: 10.1038/nm.4261 28060804

[B8] Ansaldo E. Slayden L. C. Ching K. L. Koch M. A. Wolf N. K. Plichta D. R.. (2019). Akkermansia muciniphila induces intestinal adaptive immune responses during homeostasis. Science 364 (6446), 1179–1184. doi: 10.1126/science.aaw7479 31221858 PMC6645389

[B9] Ashrafian F. Shahriary A. Behrouzi A. Moradi H. R. Keshavarz Azizi Raftar S. Lari A.. (2019). Akkermansia muciniphila-derived extracellular vesicles as a mucosal delivery vector for amelioration of obesity in mice. Front. Microbiol. 10. doi: 10.3389/fmicb.2019.02155 PMC6779730 31632356

[B10] Bae M. Cassilly C. D. Liu X. Park S. M. Tusi B. K. Chen X.. (2022). Akkermansia muciniphila phospholipid induces homeostatic immune responses. Nature 608 (7921), 168–173. doi: 10.1038/s41586-022-04985-7 35896748 PMC9328018

[B11] Baldini F. Hertel J. Sandt E. Thinnes C. C. Neuberger-Castillo L. Pavelka L.. (2020). Parkinson’s disease-associated alterations of the gut microbiome predict disease-relevant changes in metabolic functions. BMC Biol. 18 (1), 62. doi: 10.1186/s12915-020-00775-7 32517799 PMC7285525

[B12] Baranova J. Dragunas G. Botellho M. C. S. Ayub A. L. P. Bueno-Alves R. Alencar R. R.. (2021). Autism spectrum disorder: signaling pathways and prospective therapeutic targets. Cell Mol. Neurobiol. 41 (4), 619–649. doi: 10.1007/s10571-020-00882-7 32468442 PMC11448616

[B13] Barbagallo M. Dominguez L. J. (2014). Type 2 diabetes mellitus and alzheimer’s disease. World J. Diabetes 5 (6), 889–893. doi: 10.4239/wjd.v5.i6.889 25512792 PMC4265876

[B14] Barichella M. Pacchetti C. Bolliri C. Cassani E. Iorio L. Pusani C.. (2016). Probiotics and prebiotic fber for constipation associ ated with Parkinson disease: an RCT. Neurology 87 (12), 1274–1280. doi: 10.1212/WNL.0000000000003127 27543643

[B15] Batista A. F. Bodart-Santos V. De Felice F. G. Ferreira S. T. (2019). Neuroprotective actions of glucagon-like peptide-1 (GLP-1) analogues in alzheimer’s and parkinson’s diseases. CNS Drugs 33 (3), 209–223. doi: 10.1007/s40263-018-0593-6 30511349

[B16] Bedarf J. R. Hildebrand F. Coelho L. P. Sunagawa S. Bahram M. Goeser F.. (2017). Functional implications of microbial and viral gut metagenome changes in early stage l-DOPA-naive parkinson’s disease patients. Genome Med. 9 (1), 39. doi: 10.1186/s13073-017-0428-y 28449715 PMC5408370

[B17] Belzer C. De Vos W. M. (2012). Microbes inside–from diversity to function: the case of Akkermansia. ISME J. 6 (8), 1449–1458. doi: 10.1038/ismej.2012.6 22437156 PMC3401025

[B18] Benakis C. Brea D. Caballero S. Faraco G. Moore J. Murphy M.. (2016). Commensal microbiota affects ischemic stroke outcome by regulating intestinal γδ T cells. Nat. Med. 22 (5), 516–523. doi: 10.1038/nm.4068 27019327 PMC4860105

[B19] Berer K. Gerdes L. A. Cekanaviciute E. Jia X. Xiao L. Xia Z.. (2017). Gut microbiota from multiple sclerosis patients enables spontaneous autoimmune encephalomyelitis in mice. Proc. Natl. Acad. Sci. U.S.A. 114 (40), 10719–10724. doi: 10.1073/pnas.1711233114 28893994 PMC5635914

[B20] Berer K. Mues M. Koutrolos M. Rasbi Z. A. Boziki M. Johner C.. (2011). Commensal microbiota and myelin autoantigen cooperate to trigger autoimmune demyelination. Nature 479 (7374), 538–541. doi: 10.1038/nature10554 22031325

[B21] Bharadwaj P. Wijesekara N. Liyanapathirana M. Newsholme P. Ittner L. Fraser P.. (2017). The link between type 2 diabetes and neurodegeneration: roles for amyloid-β, amylin, and tau proteins. J. Alzheimers Dis. 59 (2), 421–432. doi: 10.3233/jad-161192 28269785

[B22] Bian X. Wu W. Yang L. Lv L. Wang Q. Li Y.. (2019). Administration of Akkermansia muciniphila ameliorates dextran sulfate sodium-induced ulcerative colitis in mice. Front. Microbiol. 10. doi: 10.3389/fmicb.2019.02259 PMC6779789 31632373

[B23] Borre Y. E. O’keeffe G. W. Clarke G. Stanton C. Dinan T. G. Cryan J. F. (2014). Microbiota and neurodevelopmental windows: implications for brain disorders. Trends Mol. Med. 20 (9), 509–518. doi: 10.1016/j.molmed.2014.05.002 24956966

[B24] Bosscher D. Lu Z. Janssens G. Van Caillie-Bertrand M. Robberecht H. De Rycke H.. (2001). *In vitro* availability of zinc from infant foods with increasing phytic acid contents. Br. J. Nutr. 86 (2), 241–247. doi: 10.1079/bjn2001384 11502238

[B25] Breugelmans T. Oosterlinck B. Arras W. Ceuleers H. De Man J. Hold G. L.. (2022). The role of mucins in gastrointestinal barrier function during health and disease. Lancet Gastroenterol. Hepatol. 7 (5), 455–471. doi: 10.1016/s2468-1253(21)00431-3 35397245

[B26] Buffington S. A. Di Prisco G. V. Auchtung T. A. Ajami N. J. Petrosino J. F. Costa-Mattioli M. (2016). Microbial reconstitution reverses maternal diet-induced social and synaptic deficits in offspring. Cell 165 (7), 1762–1775. doi: 10.1016/j.cell.2016.06.001 27315483 PMC5102250

[B27] Buffington S. A. Dooling S. W. Sgritta M. Noecker C. Murillo O. D. Felice D. F.. (2021). Dissecting the contribution of host genetics and the microbiome in complex behaviors. Cell 184 (7), 1740–1756.e1716. doi: 10.1016/j.cell.2021.02.009 33705688 PMC8996745

[B28] Cani P. D. Depommier C. Derrien M. Everard A. De Vos W. M. (2022). Akkermansia muciniphila: paradigm for next-generation beneficial microorganisms. Nat. Rev. Gastroenterol. Hepatol. 19 (10), 625–637. doi: 10.1038/s41575-022-00631-9 35641786

[B29] Carvalho C. Machado N. Mota P. C. Correia S. C. Cardoso S. Santos R. X.. (2013). Type 2 diabetic and alzheimer’s disease mice present similar behavioral, cognitive, and vascular anomalies. J. Alzheimers Dis. 35 (3), 623–635. doi: 10.3233/JAD-130005 23478310

[B30] Cast T. P. Boesch D. J. Smyth K. Shaw A. E. Ghebrial M. Chanda S. (2021). An autism-associated mutation impairs neuroligin-4 glycosylation and enhances excitatory synaptic transmission in human neurons. J. Neurosci. 41 (3), 392–407. doi: 10.1523/JNEUROSCI.0404-20.2020 33268543 PMC7821862

[B31] Castrén E. Kojima M. (2017). Brain-derived neurotrophic factor in mood disorders and antidepressant treatments. Neurobiol. Dis. 97 (Pt B), 119–126. doi: 10.1016/j.nbd.2016.07.010 27425886

[B32] Cekanaviciute E. Pröbstel A. K. Thomann A. Runia T. F. Casaccia P. Katz Sand I.. (2018). Multiple sclerosis-associated changes in the composition and immune functions of spore-forming bacteria. mSystems 3 (6), e00083–e00018. doi: 10.1128/mSystems.00083-18 30417113 PMC6222044

[B33] Cekanaviciute E. Yoo B. B. Runia T. F. Debelius J. W. Singh S. Nelson C. A.. (2017). Gut bacteria from multiple sclerosis patients modulate human T cells and exacerbate symptoms in mouse models. Proc. Natl. Acad. Sci. U.S.A. 114 (40), 10713–10718. doi: 10.1073/pnas.1711235114 28893978 PMC5635915

[B34] Chamorro A. Urra X. (2007). And planas, A.M. infection after acute ischemic stroke: a manifestation of brain-induced immunodepression. Stroke 38 (3), 1097–1103. doi: 10.1161/01.STR.0000258346.68966.9d 17255542

[B35] Chelakkot C. Choi Y. Kim D. K. Park H. T. Ghim J. Kwon Y.. (2018). Akkermansia muciniphila-derived extracellular vesicles influence gut permeability through the regulation of tight junctions. Exp. Mol. Med. 50 (2), e450. doi: 10.1038/emm.2017.282 29472701 PMC5903829

[B36] Chen T. Wang R. Duan Z. Yuan X. Ding Y. Feng Z.. (2021). Akkermansia muciniphila protects against psychological disorder-induced gut microbiota-mediated colonic mucosal barrier damage and aggravation of colitis. Front. Cell Infect. Microbiol. 11. doi: 10.3389/fcimb.2021.723856 PMC8551916 34722332

[B37] Chen S. Yu S. J. Li Y. Lecca D. Glotfelty E. Kim H. K.. (2018). Post-treatment with PT302, a long-acting exendin-4 sustained release formulation, reduces dopaminergic neurodegeneration in a 6-hydroxydopamine rat model of parkinson’s disease. Sci. Rep. 8 (1), 10722. doi: 10.1038/s41598-018-28449-z 30013201 PMC6048117

[B38] Cheng Y. Ling Z. Li L. (2020). The intestinal microbiota and colorectal cancer. Front. Immunol. 11. doi: 10.3389/fimmu.2020.615056 PMC7734048 33329610

[B39] Cheng Y. Liu J. Ling Z. (2021). Short-chain fatty acids-producing probiotics: a novel source of psychobiotics. Crit. Rev. Food Sci. Nutr. 62 (28), 7929–7959. doi: 10.1080/10408398.2021.1920884 33955288

[B40] Cheng J. Ringel-Kulka T. Heikamp-De Jong I. Ringel Y. Carroll I. De Vos W. M.. (2016). Discordant temporal development of bacterial phyla and the emergence of core in the fecal microbiota of young children. ISME J. 10 (4), 1002–1014. doi: 10.1038/ismej.2015.177 26430856 PMC4796939

[B41] Cheng R. Xu W. Wang J. Tang Z. Zhang M. (2021). The outer membrane protein Amuc_1100 of Akkermansia muciniphila alleviates the depression-like behavior of depressed mice induced by chronic stress. Biochem. Biophys. Res. Commun. 566, 170–176. doi: 10.1016/j.bbrc.2021.06.018 34129964

[B42] Cheng R. Zhu H. Sun Y. Hang T. Zhang M. (2022). The modified outer membrane protein Amuc_1100 of Akkermansia muciniphila improves chronic stress-induced anxiety and depression-like behavior in mice. Food Funct. 13 (20), 10748–10758. doi: 10.1039/d2fo01198k 36178497

[B43] Chevalier G. Siopi E. Guenin-Mace L. Pascal M. Laval T. Rifflet A.. (2020). Effect of gut microbiota on depressive-like behaviors in mice is mediated by the endocannabinoid system. Nat. Commun. 11 (1), 6363. doi: 10.1038/s41467-020-19931-2 33311466 PMC7732982

[B44] Cirstea M. S. Yu A. C. Golz E. Sundvick K. Kliger D. Radisavljevic N.. (2020). Microbiota composition and metabolism are associated with gut function in parkinson’s disease. Mov Disord. 35 (7), 1208–1217. doi: 10.1002/mds.28052 32357258

[B45] Clarke G. Stilling R. M. Kennedy P. J. Stanton C. Cryan J. F. Dinan T. G. (2014). Minireview: gut microbiota: the neglected endocrine organ. Mol. Endocrinol. 28 (8), 1221–1238. doi: 10.1210/me.2014-1108 24892638 PMC5414803

[B46] Collaborators, G.B.D.P.S.D (2018). Global, regional, and national burden of parkinson’s disease, 1990-2016: a systematic analysis for the global burden of disease study 2016. Lancet Neurol. 17 (11), 939–953. doi: 10.1016/S1474-4422(18)30295-3 30287051 PMC6191528

[B47] Collado M. C. Derrien M. Isolauri E. De Vos W. M. Salminen S. (2007). Intestinal integrity and Akkermansia muciniphila, a mucin-degrading member of the intestinal microbiota present in infants, adults, and the elderly. Appl. Environ. Microbiol. 73 (23), 7767–7770. doi: 10.1128/aem.01477-07 17933936 PMC2168041

[B48] Colpitts S. L. Kasper E. J. Keever A. Liljenberg C. Kirby T. Magori K.. (2017). A bidirectional association between the gut microbiota and CNS disease in a biphasic murine model of multiple sclerosis. Gut Microbes 8 (6), 561–573. doi: 10.1080/19490976.2017.1353843 28708466 PMC5730387

[B49] Coretti L. Cristiano C. Florio E. Scala G. Lama A. Keller S.. (2017). Sex-related alterations of gut microbiota composition in the BTBR mouse model of autism spectrum disorder. Sci. Rep. 7, 45356. doi: 10.1038/srep45356 28349974 PMC5368984

[B50] Coretti L. Paparo L. Riccio M. P. Amato F. Cuomo M. Natale A.. (2018). Gut microbiota features in young children with autism spectrum disorders. Front. Microbiol. 9. doi: 10.3389/fmicb.2018.03146 PMC6305749 30619212

[B51] Cox L. M. Maghzi A. H. Liu S. Tankou S. K. Dhang F. H. Willocq V.. (2021). Gut microbiome in progressive multiple sclerosis. Ann. Neurol. 89 (6), 1195–1211. doi: 10.1002/ana.26084 33876477 PMC8132291

[B52] Craft S. (2005). Insulin resistance syndrome and alzheimer’s disease: age- and obesity-related effects on memory, amyloid, and inflammation. Neurobiol. Aging 26 Suppl 1, 65–69. doi: 10.1016/j.neurobiolaging.2005.08.021 16266773

[B53] Dalile B. Van Oudenhove L. Vervliet B. Verbeke K. (2019). The role of short-chain fatty acids in microbiota-gut-brain communication. Nat. Rev. Gastroenterol. Hepatol. 16 (8), 461–478. doi: 10.1038/s41575-019-0157-3 31123355

[B54] Dao M. C. Everard A. Aron-Wisnewsky J. Sokolovska N. Prifti E. Verger E. O.. (2016). Akkermansia muciniphila and improved metabolic health during a dietary intervention in obesity: relationship with gut microbiome richness and ecology. Gut 65 (3), 426–436. doi: 10.1136/gutjnl-2014-308778 26100928

[B55] De Angelis M. Piccolo M. Vannini L. Siragusa S. De Giacomo A. Serrazzanetti D. I.. (2013). Fecal microbiota and metabolome of children with autism and pervasive developmental disorder not otherwise specified. PloS One 8 (10), e76993. doi: 10.1371/journal.pone.0076993 24130822 PMC3793965

[B56] De Felice F. G. (2013). Connecting type 2 diabetes to alzheimer’s disease. Expert Rev. Neurother 13 (12), 1297–1299. doi: 10.1586/14737175.2013.864824 24236899

[B57] Degos V. Favrais G. Kaindl A. M. Peineau S. Guerrot A. M. Verney C.. (2010). Inflammation processes in perinatal brain damage. J. Neural Transm (Vienna) 117 (8), 1009–1017. doi: 10.1007/s00702-010-0411-x 20473533

[B58] De Magistris L. Familiari V. Pascotto A. Sapone A. Frolli A. Iardino P.. (2010). Alterations of the intestinal barrier in patients with autism spectrum disorders and in their first-degree relatives. J. Pediatr. Gastroenterol. Nutr. 51 (4), 418–424. doi: 10.1097/MPG.0b013e3181dcc4a5 20683204

[B59] Depommier C. Everard A. Druart C. Plovier H. Van Hul M. Vieira-Silva S.. (2019). Supplementation with Akkermansia muciniphila in overweight and obese human volunteers: a proof-of-concept exploratory study. Nat. Med. 25 (7), 1096–1103. doi: 10.1038/s41591-019-0495-2 31263284 PMC6699990

[B60] Derrien M. Belzer C. De Vos W. M. (2017). Akkermansia muciniphila and its role in regulating host functions. Microb. Pathog. 106, 171–181. doi: 10.1016/j.micpath.2016.02.005 26875998

[B61] Derrien M. Van Baarlen P. Hooiveld G. Norin E. Muller M. De Vos W. M. (2011). Modulation of mucosal immune response, tolerance, and proliferation in mice colonized by the mucin-degrader Akkermansia muciniphila. Front. Microbiol. 2. doi: 10.3389/fmicb.2011.00166 PMC3153965 21904534

[B62] Derrien M. Van Passel M. W. Van De Bovenkamp J. H. Schipper R. G. De Vos W. M. Dekker J. (2010). Mucin-bacterial interactions in the human oral cavity and digestive tract. Gut Microbes 1 (4), 254–268. doi: 10.4161/gmic.1.4.12778 21327032 PMC3023607

[B63] Derrien M. Vaughan E. E. Plugge C. M. De Vos W. M. (2004). Akkermansia muciniphila gen. nov., sp. nov., a human intestinal mucin-degrading bacterium. Int. J. Syst. Evol. Microbiol. 54 (Pt 5), 1469–1476. doi: 10.1099/ijs.0.02873-0 15388697

[B64] De Theije C. G. Wopereis H. Ramadan M. Van Eijndthoven T. Lambert J. Knol J.. (2014). Altered gut microbiota and activity in a murine model of autism spectrum disorders. Brain Behav. Immun. 37, 197–206. doi: 10.1016/j.bbi.2013.12.005 24333160

[B65] Dickson R. P. Erb-Downward J. R. Freeman C. M. Mccloskey L. Falkowski N. R. Huffnagle G. B.. (2017). Bacterial topography of the healthy human lower respiratory tract. mBio 8 (1), e02287–e02216. doi: 10.1128/mBio.02287-16 28196961 PMC5312084

[B66] Ding Y. Bu F. Chen T. Shi G. Yuan X. Feng Z.. (2021). A next-generation probiotic: Akkermansia muciniphila ameliorates chronic stress-induced depressive-like behavior in mice by regulating gut microbiota and metabolites. Appl. Microbiol. Biotechnol. 105 (21-22), 8411–8426. doi: 10.1007/s00253-021-11622-2 34617139

[B67] Dodiya H. B. Forsyth C. B. Voigt R. M. Engen P. A. Patel J. Shaikh M.. (2020). Chronic stress-induced gut dysfunction exacerbates parkinson’s disease phenotype and pathology in a rotenone-induced mouse model of parkinson’s disease. Neurobiol. Dis. 135, 104352. doi: 10.1016/j.nbd.2018.12.012 30579705

[B68] Duman R. S. Monteggia L. M. (2006). A neurotrophic model for stress-related mood disorders. Biol. Psychiatry 59 (12), 1116–1127. doi: 10.1016/j.biopsych.2006.02.013 16631126

[B69] Ellis T. N. Kuehn M. J. (2010). Virulence and immunomodulatory roles of bacterial outer membrane vesicles. Microbiol. Mol. Biol. Rev. 74 (1), 81–94. doi: 10.1128/MMBR.00031-09 20197500 PMC2832350

[B70] Everard A. Belzer C. Geurts L. Ouwerkerk J. P. Druart C. Bindels L. B.. (2013). Cross-talk between Akkermansia muciniphila and intestinal epithelium controls diet-induced obesity. Proc. Natl. Acad. Sci. U.S.A. 110 (22), 9066–9071. doi: 10.1073/pnas.1219451110 23671105 PMC3670398

[B71] Fang X. Li F. J. Hong D. J. (2021). Potential role of Akkermansia muciniphila in parkinson’s disease and other Neurological/Autoimmune diseases. Curr. Med. Sci. 41 (6), 1172–1177. doi: 10.1007/s11596-021-2464-5 34893951

[B72] Feldman G. J. Mullin J. M. Ryan M. P. (2005). Occludin: structure, function and regulation. Adv. Drug Delivery Rev. 57 (6), 883–917. doi: 10.1016/j.addr.2005.01.009 15820558

[B73] Ferguson B. J. Marler S. Altstein L. L. Lee E. B. Akers J. Sohl K.. (2017). Psychophysiological associations with gastrointestinal symptomatology in autism spectrum disorder. Autism Res. 10 (2), 276–288. doi: 10.1002/aur.1646 27321113 PMC5526214

[B74] Finegold S. M. Dowd S. E. Gontcharova V. Liu C. Henley K. E. Wolcott R. D.. (2010). Pyrosequencing study of fecal microflora of autistic and control children. Anaerobe 16 (4), 444–453. doi: 10.1016/j.anaerobe.2010.06.008 20603222

[B75] Foster J. A. (2022). Modulating brain function with microbiota. Science 376 (6596), 936–937. doi: 10.1126/science.abo4220 35617384

[B76] Foster J. A. Rinaman L. Cryan J. F. (2017). Stress & the gut-brain axis: regulation by the microbiome. Neurobiol. Stress 7, 124–136. doi: 10.1016/j.ynstr.2017.03.001 29276734 PMC5736941

[B77] Freude S. Plum L. Schnitker J. Leeser U. Udelhoven M. Krone W.. (2005). Peripheral hyperinsulinemia promotes tau phosphorylation *in vivo* . Diabetes 54 (12), 3343–3348. doi: 10.2337/diabetes.54.12.3343 16306348

[B78] Fujisaka S. Usui I. Nawaz A. Igarashi Y. Okabe K. Furusawa Y.. (2020). Bofutsushosan improves gut barrier function with a bloom of Akkermansia muciniphila and improves glucose metabolism in mice with diet-induced obesity. Sci. Rep. 10 (1), 5544. doi: 10.1038/s41598-020-62506-w 32218475 PMC7099031

[B79] Ganesh B. P. Klopfleisch R. Loh G. Blaut M. (2013). Commensal Akkermansia muciniphila exacerbates gut inflammation in salmonella typhimurium-infected gnotobiotic mice. PloS One 8 (9), e74963. doi: 10.1371/journal.pone.0074963 24040367 PMC3769299

[B80] Gómez-Gallego C. Pohl S. Salminen S. De Vos W. M. Kneifel W. (2016). Akkermansia muciniphila: a novel functional microbe with probiotic properties. Benef Microbes 7 (4), 571–584. doi: 10.3920/bm2016.0009 27291403

[B81] Gondalia S. V. Palombo E. A. Knowles S. R. Cox S. B. Meyer D. Austin D. W. (2012). Molecular characterisation of gastrointestinal microbiota of children with autism (with and without gastrointestinal dysfunction) and their neurotypical siblings. Autism Res. 5 (6), 419–427. doi: 10.1002/aur.1253 22997101

[B82] Goo N. Bae H. J. Park K. Kim J. Jeong Y. Cai M.. (2020). The effect of fecal microbiota transplantation on autistic-like behaviors in Fmr1 KO mice. Life Sci. 262, 118497. doi: 10.1016/j.lfs.2020.118497 32987062

[B83] Gordon N. Goelman G. (2016). Understanding alterations in serotonin connectivity in a rat model of depression within the monoamine-deficiency and the hippocampal-neurogenesis frameworks. Behav. Brain Res. 296, 141–148. doi: 10.1016/j.bbr.2015.09.013 26367472

[B84] Grajeda-Iglesias C. Durand S. Daillère R. Iribarren K. Lemaitre F. Derosa L.. (2021). Oral administration of Akkermansia muciniphila elevates systemic antiaging and anticancer metabolites. Aging (Albany NY) 13 (5), 6375–6405. doi: 10.18632/aging.202739 33653967 PMC7993698

[B85] Grander C. Adolph T. E. Wieser V. Lowe P. Wrzosek L. Gyongyosi B.. (2018). Recovery of ethanol-induced Akkermansia muciniphila depletion ameliorates alcoholic liver disease. Gut 67 (5), 891–901. doi: 10.1136/gutjnl-2016-313432 28550049

[B86] Greer R. L. Dong X. Moraes A. C. Zielke R. A. Fernandes G. R. Peremyslova E.. (2016). Akkermansia muciniphila mediates negative effects of IFNγ on glucose metabolism. Nat. Commun. 7, 13329. doi: 10.1038/ncomms13329 27841267 PMC5114536

[B87] Grenham S. Clarke G. Cryan J. F. Dinan T. G. (2011). Brain-gut-microbe communication in health and disease. Front. Physiol. 2. doi: 10.3389/fphys.2011.00094 PMC3232439 22162969

[B88] Griciuc A. Tanzi R. E. (2021). The role of innate immune genes in alzheimer’s disease. Curr. Opin. Neurol. 34 (2), 228–236. doi: 10.1097/WCO.0000000000000911 33560670 PMC7954128

[B89] Guo D. Park C. Li Y. Li B. Yang Q. Deng Y.. (2022). Akkermansia muciniphila ameliorates depressive disorders in a murine alcohol-LPS (mALPS) model. Food Funct. 13 (24), 12766–12776. doi: 10.1039/d2fo01478e 36416490

[B90] Gurung M. Li Z. You H. Rodrigues R. Jump D. B. Morgun A.. (2020). Role of gut microbiota in type 2 diabetes pathophysiology. EBioMedicine 51, 102590. doi: 10.1016/j.ebiom.2019.11.051 31901868 PMC6948163

[B91] Hagberg H. Mallard C. (2005). Effect of inflammation on central nervous system development and vulnerability. Curr. Opin. Neurol. 18 (2), 117–123. doi: 10.1097/01.wco.0000162851.44897.8f 15791140

[B92] Hänninen A. Toivonen R. Pöysti S. Belzer C. Plovier H. Ouwerkerk J. P.. (2018). Akkermansia muciniphila induces gut microbiota remodelling and controls islet autoimmunity in NOD mice. Gut 67 (8), 1445–1453. doi: 10.1136/gutjnl-2017-314508 29269438

[B93] Harach T. Marungruang N. Duthilleul N. Cheatham V. Mc Coy K. D. Frisoni G.. (2017). Reduction of abeta amyloid pathology in APPPS1 transgenic mice in the absence of gut microbiota. Sci. Rep. 7, 41802. doi: 10.1038/srep41802 28176819 PMC5297247

[B94] Hill-Burns E. M. Debelius J. W. Morton J. T. Wissemann W. T. Lewis M. R. Wallen Z. D.. (2017). Parkinson’s disease and parkinson’s disease medications have distinct signatures of the gut microbiome. Mov Disord. 32 (5), 739–749. doi: 10.1002/mds.26942 28195358 PMC5469442

[B95] Hing B. Sathyaputri L. Potash J. B. (2018). A comprehensive review of genetic and epigenetic mechanisms that regulate BDNF expression and function with relevance to major depressive disorder. Am. J. Med. Genet. B Neuropsychiatr. Genet. 177 (2), 143–167. doi: 10.1002/ajmg.b.32616 29243873

[B96] Holst J. J. Burcelin R. Nathanson E. (2011). Neuroprotective properties of GLP-1: theoretical and practical applications. Curr. Med. Res. Opin. 27 (3), 547–558. doi: 10.1185/03007995.2010.549466 21222567

[B97] Hossmann K. A. (2006). Pathophysiology and therapy of experimental stroke. Cell Mol. Neurobiol. 26 (7-8), 1057–1083. doi: 10.1007/s10571-006-9008-1 16710759 PMC11520628

[B98] Hou Y. Li X. Liu C. Zhang M. Zhang X. Ge S.. (2021). Neuroprotective effects of short-chain fatty acids in MPTP induced mice model of parkinson’s disease. Exp. Gerontol 150, 111376. doi: 10.1016/j.exger.2021.111376 33905875

[B99] Hsiao E. Y. Mcbride S. W. Hsien S. Sharon G. Hyde E. R. Mccue T.. (2013). Microbiota modulate behavioral and physiological abnormalities associated with neurodevelopmental disorders. Cell 155 (7), 1451–1463. doi: 10.1016/j.cell.2013.11.024 24315484 PMC3897394

[B100] Hu X. Zhao Y. Yang Y. Gong W. Sun X. Yang L.. (2020). Akkermansia muciniphila improves host defense against influenza virus infection. Front. Microbiol. 11. doi: 10.3389/fmicb.2020.586476 PMC7884316 33603716

[B101] Jeon H. Bae C. H. Lee Y. Kim H. Y. Kim S. (2021). Korean Red ginseng suppresses 1-methyl-4-phenyl-1,2,3,6-tetrahydropyridine-induced inflammation in the substantia nigra and colon. Brain Behav. Immun. 94, 410–423. doi: 10.1016/j.bbi.2021.02.028 33662500

[B102] Ji W. Zhu Y. Kan P. Cai Y. Wang Z. Wu Z.. (2017). Analysis of intestinal microbial communities of cerebral infarction and ischemia patients based on high throughput sequencing technology and glucose and lipid metabolism. Mol. Med. Rep. 16 (4), 5413–5417. doi: 10.3892/mmr.2017.7227 28849032 PMC5647104

[B103] Jiang H. Ling Z. Zhang Y. Mao H. Ma Z. Yin Y.. (2015). Altered fecal microbiota composition in patients with major depressive disorder. Brain Behav. Immun. 48, 186–194. doi: 10.1016/j.bbi.2015.03.016 25882912

[B104] Johansson M. E. Larsson J. M. Hansson G. C. (2011). The two mucus layers of colon are organized by the MUC2 mucin, whereas the outer layer is a legislator of host-microbial interactions. Proc. Natl. Acad. Sci. U.S.A. 108 Suppl 1, 4659–4665. doi: 10.1073/pnas.1006451107 20615996 PMC3063600

[B105] Joseph J. Depp C. Shih P. B. Cadenhead K. S. Schmid-Schönbein G. (2017). Modified Mediterranean diet for enrichment of short chain fatty acids: potential adjunctive therapeutic to target immune and metabolic dysfunction in schizophrenia? Front. Neurosci. 11. doi: 10.3389/fnins.2017.00155 PMC5366345 28396623

[B106] Kang D. W. Park J. G. Ilhan Z. E. Wallstrom G. Labaer J. Adams J. B.. (2013). Reduced incidence of prevotella and other fermenters in intestinal microflora of autistic children. PloS One 8 (7), e68322. doi: 10.1371/journal.pone.0068322 23844187 PMC3700858

[B107] Keshavarz Azizi Raftar S. Ashrafian F. Yadegar A. Lari A. Moradi H. R. Shahriary A.. (2021). The protective effects of live and pasteurized Akkermansia muciniphila and its extracellular vesicles against HFD/CCl4-induced liver injury. Microbiol. Spectr. 9 (2), e0048421. doi: 10.1128/Spectrum.00484-21 34549998 PMC8557882

[B108] Keshavarzian A. Green S. J. Engen P. A. Voigt R. M. Naqib A. Forsyth C. B.. (2015). Colonic bacterial composition in parkinson’s disease. Mov Disord. 30 (10), 1351–1360. doi: 10.1002/mds.26307 26179554

[B109] Khedr E. M. Omeran N. Karam-Allah Ramadan H. Ahmed G. K. Abdelwarith A. M. (2022). Alteration of gut microbiota in alzheimer’s disease and their relation to the cognitive impairment. J. Alzheimers Dis. 88 (3), 1103–1114. doi: 10.3233/JAD-220176 35754271

[B110] Kia D. A. Zhang D. Guelfi S. Manzoni C. Hubbard L. Reynolds R. H.. (2021). Identification of candidate Parkinson disease genes by integrating genome-wide association study, expression, and epigenetic data sets. JAMA Neurol. 78 (4), 464–472. doi: 10.1001/jamaneurol.2020.5257 33523105 PMC7851759

[B111] Kim Y. S. Choi E. J. Lee W. H. Choi S. J. Roh T. Y. Park J.. (2013). Extracellular vesicles, especially derived from gram-negative bacteria, in indoor dust induce neutrophilic pulmonary inflammation associated with both Th1 and Th17 cell responses. Clin. Exp. Allergy 43 (4), 443–454. doi: 10.1111/cea.12085 23517040

[B112] Kim C. Ho D. H. Suk J. E. You S. Michael S. Kang J.. (2013). Neuron-released oligomeric alpha-synuclein is an endogenous agonist of TLR2 for paracrine activation of microglia. Nat. Commun. 4, 1562. doi: 10.1038/ncomms2534 23463005 PMC4089961

[B113] Kim S. Kim H. Yim Y. S. Ha S. Atarashi K. Tan T. G.. (2017). Maternal gut bacteria promote neurodevelopmental abnormalities in mouse offspring. Nature 549 (7673), 528–532. doi: 10.1038/nature23910 28902840 PMC5870873

[B114] Knopman D. S. Amieva H. Petersen R. C. Chetelat G. Holtzman D. M. Hyman B. T.. (2021). Alzheimer Disease. Nat. Rev. Dis. Primers 7 (1), 33. doi: 10.1038/s41572-021-00269-y 33986301 PMC8574196

[B115] Konig J. Wells J. Cani P. D. Garcia-Rodenas C. L. Macdonald T. Mercenier A.. (2016). Human intestinal barrier function in health and disease. Clin. Transl. Gastroenterol. 7 (10), e196. doi: 10.1038/ctg.2016.54 27763627 PMC5288588

[B116] Kostopoulos I. Elzinga J. Ottman N. Klievink J. T. Blijenberg B. Aalvink S.. (2020). Akkermansia muciniphila uses human milk oligosaccharides to thrive in the early life conditions *in vitro* . Sci. Rep. 10 (1), 14330. doi: 10.1038/s41598-020-71113-8 32868839 PMC7459334

[B117] Koutrolos M. Berer K. Kawakami N. Wekerle H. Krishnamoorthy G. (2014). Treg cells mediate recovery from EAE by controlling effector T cell proliferation and motility in the CNS. Acta Neuropathol. Commun. 2, 163. doi: 10.1186/s40478-014-0163-1 25476447 PMC4268825

[B118] Kozhieva M. Naumova N. Alikina T. Boyko A. Vlassov V. (2019). And kabilov, M.R. primary progressive multiple sclerosis in a Russian cohort: relationship with gut bacterial diversity. BMC Microbiol. 19 (1), 309. doi: 10.1186/s12866-019-1685-2 31888483 PMC6937728

[B119] Kratsman N. Getselter D. Elliott E. (2016). Sodium butyrate attenuates social behavior deficits and modifies the transcription of inhibitory/excitatory genes in the frontal cortex of an autism model. Neuropharmacology 102, 136–145. doi: 10.1016/j.neuropharm.2015.11.003 26577018

[B120] Kuehn M. J. Kesty N. C. (2005). Bacterial outer membrane vesicles and the host-pathogen interaction. Genes Dev. 19 (22), 2645–2655. doi: 10.1101/gad.1299905 16291643

[B121] Kuhlmann J. Andreasson U. Pannee J. Bjerke M. Portelius E. Leinenbach A.. (2017). CSF Abeta1-42 - an excellent but complicated alzheimer’s biomarker - a route to standardisation. Clin. Chim. Acta 467, 27–33. doi: 10.1016/j.cca.2016.05.014 27216941

[B122] Kushak R. I. Winter H. S. Buie T. M. Cox S. B. Phillips C. D. Ward N. L. (2017). Analysis of the duodenal microbiome in autistic individuals: association with carbohydrate digestion. J. Pediatr. Gastroenterol. Nutr. 64 (5), e110–e116. doi: 10.1097/mpg.0000000000001458 27811623

[B123] Lee Y. K. Menezes J. S. Umesaki Y. Mazmanian S. K. (2011). Proinflammatory T-cell responses to gut microbiota promote experimental autoimmune encephalomyelitis. Proc. Natl. Acad. Sci. U.S.A. 108 Suppl 1 (Suppl 1), 4615–4622. doi: 10.1073/pnas.1000082107 20660719 PMC3063590

[B124] Li X. Fan X. Yuan X. Pang L. Hu S. Wang Y.. (2021). The role of butyric acid in treatment response in drug-naïve first episode schizophrenia. Front. Psychiatry 12. doi: 10.3389/fpsyt.2021.724664 PMC8421030 34497548

[B125] Li Z. Lai J. Zhang P. Ding J. Jiang J. Liu C.. (2022). Multi-omics analyses of serum metabolome, gut microbiome and brain function reveal dysregulated microbiota-gut-brain axis in bipolar depression. Mol. Psychiatry 27 (10), 4123–4135. doi: 10.1038/s41380-022-01569-9 35444255

[B126] Li J. Lin S. Vanhoutte P. M. Woo C. W. Xu A. (2016). Akkermansia muciniphila protects against atherosclerosis by preventing metabolic endotoxemia-induced inflammation in apoe-/- mice. Circulation 133 (24), 2434–2446. doi: 10.1161/circulationaha.115.019645 27143680

[B127] Li N. Wang X. Sun C. Wu X. Lu M. Si Y.. (2019). Change of intestinal microbiota in cerebral ischemic stroke patients. BMC Microbiol. 19 (1), 191. doi: 10.1186/s12866-019-1552-1 31426765 PMC6700817

[B128] Li J. Zhao F. Wang Y. Chen J. Tao J. Tian G.. (2017). Gut microbiota dysbiosis contributes to the development of hypertension. Microbiome 5 (1), 14. doi: 10.1186/s40168-016-0222-x 28143587 PMC5286796

[B129] Liang S. Wu X. Hu X. Wang T. Jin F. (2018). Recognizing depression from the microbiota(-)Gut(-)Brain axis. Int. J. Mol. Sci. 19 (6), 1592. doi: 10.3390/ijms19061592 29843470 PMC6032096

[B130] Linden A. G. Hwa S. L. Choi Y. Fang F. Fukasawa M. Uyeda K.. (2018). Interplay between ChREBP and SREBP-1c coordinates postprandial glycolysis and lipogenesis in livers of mice. J. Lipid Res. 59 (3), 475–487. doi: 10.1194/jlr.M081836 29335275 PMC5832931

[B131] Ling Z. Cheng Y. Chen F. Yan X. Liu X. Shao L.. (2022). Changes in fecal microbiota composition and the cytokine expression profile in school-aged children with depression: a case-control study. Front. Immunol. 13. doi: 10.3389/fimmu.2022.964910 PMC9437487 36059521

[B132] Ling Z. Cheng Y. Yan X. Shao L. Liu X. Zhou D.. (2020a). Alterations of the fecal microbiota in Chinese patients with multiple sclerosis. Front. Immunol. 11. doi: 10.3389/fimmu.2020.590783 PMC7772405 33391265

[B133] Ling Z. X. Xiao H. Chen W. (2022). Gut microbiome: the cornerstone of life and health. Adv. Gut Microbiome Res. 2022, 9894812. doi: 10.1155/2022/9894812

[B134] Ling Z. Zhu M. Yan X. Cheng Y. Shao L. Liu X.. (2020b). Structural and functional dysbiosis of fecal microbiota in Chinese patients with alzheimer’s disease. Front. Cell Dev. Biol. 8. doi: 10.3389/fcell.2020.634069 PMC7889981 33614635

[B135] Liu X. Cui Y. Zhang Y. Xiang G. Yu M. Wang X.. (2022). Rescue of social deficits by early-life melatonin supplementation through modulation of gut microbiota in a murine model of autism. BioMed. Pharmacother. 156, 113949. doi: 10.1016/j.biopha.2022.113949 36411634

[B136] Liu R. Hong J. Xu X. Feng Q. Zhang D. Gu Y.. (2017). Gut microbiome and serum metabolome alterations in obesity and after weight-loss intervention. Nat. Med. 23 (7), 859–868. doi: 10.1038/nm.4358 28628112

[B137] Liu F. Horton-Sparks K. Hull V. Li R. W. Martínez-Cerdeño V. (2018). The valproic acid rat model of autism presents with gut bacterial dysbiosis similar to that in human autism. Mol. Autism 9, 61. doi: 10.1186/s13229-018-0251-3 30555669 PMC6288876

[B138] Liu Q. Lu W. Tian F. Zhao J. Zhang H. Hong K.. (2021). Akkermansia muciniphila exerts strain-specific effects on DSS-induced ulcerative colitis in mice. Front. Cell Infect. Microbiol. 11. doi: 10.3389/fcimb.2021.698914 PMC8371549 34422681

[B139] Liu S. Rezende R. M. Moreira T. G. Tankou S. K. Cox L. M. Wu M.. (2019). Oral administration of miR-30d from feces of MS patients suppresses MS-like symptoms in mice by expanding Akkermansia muciniphila. Cell Host Microbe 26 (6), 779–794.e778. doi: 10.1016/j.chom.2019.10.008 31784260 PMC6948921

[B140] Liu P. S. Wang H. Li X. Chao T. Teav T. Christen S.. (2017). Alpha-ketoglutarate orchestrates macrophage activation through metabolic and epigenetic reprogramming. Nat. Immunol. 18 (9), 985–994. doi: 10.1038/ni.3796 28714978

[B141] Liu Y. Yang M. Tang L. Wang F. Huang S. Liu S.. (2022). TLR4 regulates RORγt(+) regulatory T-cell responses and susceptibility to colon inflammation through interaction with Akkermansia muciniphila. Microbiome 10 (1), 98. doi: 10.1186/s40168-022-01296-x 35761415 PMC9235089

[B142] Ma X. Guo Y. Xu J. Wang X. Dong S. Gao Y.. (2022). Effects of distinct n-6 to n-3 polyunsaturated fatty acid ratios on insulin resistant and AD-like phenotypes in high-fat diets-fed APP/PS1 mice. Food Res. Int. 162 (Pt B), 112207. doi: 10.1016/j.foodres.2022.112207 36461380

[B143] Mcgaughey K. D. Yilmaz-Swenson T. Elsayed N. M. Cruz D. A. Rodriguiz R. M. Kritzer M. D.. (2019). Relative abundance of Akkermansia spp. and other bacterial phylotypes correlates with anxiety- and depressive-like behavior following social defeat in mice. Sci. Rep. 9 (1), 3281. doi: 10.1038/s41598-019-40140-5 30824791 PMC6397238

[B144] Mcginley A. M. Edwards S. C. Raverdeau M. Mills K. H. G. (2018). Th17 cells, γδ T cells and their interplay in EAE and multiple sclerosis. J. Autoimmun S0896-8411 (18), 30007–30006. doi: 10.1016/j.jaut.2018.01.001 29395738

[B145] Mlynarska E. Gadzinowska J. Tokarek J. Forycka J. Szuman A. Franczyk B.. (2022). The role of the microbiome-Brain-Gut axis in the pathogenesis of depressive disorder. Nutrients 14 (9), 1921. doi: 10.3390/nu14091921 35565888 PMC9105444

[B146] Mollenhauer B. Caf. V. A. (2022). Toward preventing parkinson’s disease. Science 377 (6608), 818–819. doi: 10.1126/science.add7162 35981039

[B147] Na Y. R. Stakenborg M. Seok S. H. Matteoli G. (2019). Macrophages in intestinal inflammation and resolution: a potential therapeutic target in IBD. Nat. Rev. Gastroenterol. Hepatol. 16 (9), 531–543. doi: 10.1038/s41575-019-0172-4 31312042

[B148] Nagpal R. Neth B. J. Wang S. Craft S. Yadav H. (2019). Modified Mediterranean-ketogenic diet modulates gut microbiome and short-chain fatty acids in association with alzheimer’s disease markers in subjects with mild cognitive impairment. EBioMedicine 47, 529–542. doi: 10.1016/j.ebiom.2019.08.032 31477562 PMC6796564

[B149] Newell C. Bomhof M. R. Reimer R. A. Hittel D. S. Rho J. M. Shearer J. (2016). Ketogenic diet modifies the gut microbiota in a murine model of autism spectrum disorder. Mol. Autism 7 (1), 37. doi: 10.1186/s13229-016-0099-3 27594980 PMC5009541

[B150] Nishimura S. Manabe I. Nagasaki M. Eto K. Yamashita H. Ohsugi M.. (2009). CD8+ effector T cells contribute to macrophage recruitment and adipose tissue inflammation in obesity. Nat. Med. 15 (8), 914–920. doi: 10.1038/nm.1964 19633658

[B151] Oh J. Vidal-Jordana A. Montalban X. (2018). Multiple sclerosis: clinical aspects. Curr. Opin. Neurol. 31 (6), 752–759. doi: 10.1097/WCO.0000000000000622 30300239

[B152] O’mahony S. M. Clarke G. Borre Y. E. Dinan T. G. Cryan J. F. (2015). Serotonin, tryptophan metabolism and the brain-gut-microbiome axis. Behav. Brain Res. 277, 32–48. doi: 10.1016/j.bbr.2014.07.027 25078296

[B153] Ottman N. Reunanen J. Meijerink M. Pietila T. E. Kainulainen V. Klievink J.. (2017). Pili-like proteins of Akkermansia muciniphila modulate host immune responses and gut barrier function. PloS One 12 (3), e0173004. doi: 10.1371/journal.pone.0173004 28249045 PMC5332112

[B154] Ou Z. Deng L. Lu Z. Wu F. Liu W. Huang D.. (2020). Protective effects of Akkermansia muciniphila on cognitive deficits and amyloid pathology in a mouse model of alzheimer’s disease. Nutr. Diabetes 10 (1), 12. doi: 10.1038/s41387-020-0115-8 32321934 PMC7176648

[B155] Park Y. S. Kim S. H. Park J. W. Kho Y. Seok P. R. Shin J. H.. (2020). Melatonin in the colon modulates intestinal microbiota in response to stress and sleep deprivation. Intest Res. 18 (3), 325–336. doi: 10.5217/ir.2019.00093 32564539 PMC7385569

[B156] Parr E. Ferdinand P. Roffe C. (2017). Management of acute stroke in the older person. Geriatrics (Basel) 2 (3), 27. doi: 10.3390/geriatrics2030027 31011037 PMC6371128

[B157] Payahoo L. Khajebishak Y. Alivand M. R. Soleimanzade H. Alipour S. Barzegari A.. (2019). Investigation the effect of oleoylethanolamide supplementation on the abundance of Akkermansia muciniphila bacterium and the dietary intakes in people with obesity: a randomized clinical trial. Appetite 141, 104301. doi: 10.1016/j.appet.2019.05.032 31132422

[B158] Persico A. M. Ricciardello A. Cucinotta F. (2019). The psychopharmacology of autism spectrum disorder and rett syndrome. Handb. Clin. Neurol. 165, 391–414. doi: 10.1016/B978-0-444-64012-3.00024-1 31727226

[B159] Pettigrew M. M. Gent J. F. Kong Y. Wade M. Gansebom S. Bramley A. M.. (2016). Association of sputum microbiota profiles with severity of community-acquired pneumonia in children. BMC Infect. Dis. 16, 317. doi: 10.1186/s12879-016-1670-4 27391033 PMC4939047

[B160] Pistollato F. Sumalla Cano S. Elio I. Masias Vergara M. Giampieri F. Battino M. (2016). Role of gut microbiota and nutrients in amyloid formation and pathogenesis of Alzheimer disease. Nutr. Rev. 74 (10), 624–634. doi: 10.1093/nutrit/nuw023 27634977

[B161] Plovier H. Everard A. Druart C. Depommier C. Van Hul M. Geurts L.. (2017). A purified membrane protein from Akkermansia muciniphila or the pasteurized bacterium improves metabolism in obese and diabetic mice. Nat. Med. 23 (1), 107–113. doi: 10.1038/nm.4236 27892954

[B162] Pluta R. Ulamek-Koziol M. Kocki J. Bogucki J. Januszewski S. Bogucka-Kocka A.. (2020). Expression of the tau protein and amyloid protein precursor processing genes in the CA3 area of the hippocampus in the ischemic model of alzheimer’s disease in the rat. Mol. Neurobiol. 57 (2), 1281–1290. doi: 10.1007/s12035-019-01799-z 31713815 PMC7031177

[B163] Psichas A. Sleeth M. L. Murphy K. G. Brooks L. Bewick G. A. Hanyaloglu A. C.. (2015). The short chain fatty acid propionate stimulates GLP-1 and PYY secretion via free fatty acid receptor 2 in rodents. Int. J. Obes. (Lond) 39 (3), 424–429. doi: 10.1038/ijo.2014.153 25109781 PMC4356745

[B164] Qian Y. Yang X. Xu S. Huang P. Li B. Du J.. (2020). Gut metagenomics-derived genes as potential biomarkers of parkinson’s disease. Brain 143 (8), 2474–2489. doi: 10.1093/brain/awaa201 32844199

[B165] Qin J. Li R. Raes J. Arumugam M. Burgdorf K. S. Manichanh C.. (2010). A human gut microbial gene catalogue established by metagenomic sequencing. Nature 464 (7285), 59–65. doi: 10.1038/nature08821 20203603 PMC3779803

[B166] Radenovic L. Nenadic M. Ułamek-Kozioł M. Januszewski S. Czuczwar S. J. Andjus P. R.. (2020). Heterogeneity in brain distribution of activated microglia and astrocytes in a rat ischemic model of Alzheimer’s disease after 2 years of survival. Aging (Albany NY) 12 (12), 12251–12267. doi: 10.18632/aging.103411 32501292 PMC7343500

[B167] Rakhit S. Clark C. J. O’shaughnessy C T. Morris B. J. (2005). N-methyl-D-aspartate and brain-derived neurotrophic factor induce distinct profiles of extracellular signal-regulated kinase, mitogen- and stress-activated kinase, and ribosomal s6 kinase phosphorylation in cortical neurons. Mol. Pharmacol. 67 (4), 1158–1165. doi: 10.1124/mol.104.005447 15625280

[B168] Ransohoff R. M. Hafler D. A. Lucchinetti C. F. (2015). Multiple sclerosis-a quiet revolution. Nat. Rev. Neurol. 11 (3), 134–142. doi: 10.1038/nrneurol.2015.14 25686758 PMC4556342

[B169] Reich S. G. Savitt J. M. (2019). Parkinson’s disease. Med. Clin. North Am. 103 (2), 337–350. doi: 10.1016/j.mcna.2018.10.014 30704685

[B170] Reunanen J. Kainulainen V. Huuskonen L. Ottman N. Belzer C. Huhtinen H.. (2015). Akkermansia muciniphila adheres to enterocytes and strengthens the integrity of the epithelial cell layer. Appl. Environ. Microbiol. 81 (11), 3655–3662. doi: 10.1128/AEM.04050-14 25795669 PMC4421065

[B171] Ring A. Kim Y. M. Kahn M. (2014). Wnt/catenin signaling in adult stem cell physiology and disease. Stem Cell Rev. Rep. 10 (4), 512–525. doi: 10.1007/s12015-014-9515-2 24825509 PMC4294579

[B172] Ruiz N. Del Ángel D. S. Olguín H. J. Silva M. L. (2018). Neuroprogression: the hidden mechanism of depression. Neuropsychiatr. Dis. Treat 14, 2837–2845. doi: 10.2147/ndt.S177973 30464468 PMC6214587

[B173] Ryuk J. A. Ko B. S. Moon N. R. Park S. (2022). Protection against neurological symptoms by consuming corn silk water extract in artery-occluded gerbils with reducing oxidative stress, inflammation, and post-stroke hyperglycemia through the gut-brain axis. Antioxidants (Basel) 11 (1), 168. doi: 10.3390/antiox11010168 35052672 PMC8773031

[B174] Sauer A. K. Bockmann J. Steinestel K. Boeckers T. M. Grabrucker A. M. (2019). Altered intestinal morphology and microbiota composition in the autism spectrum disorders associated SHANK3 mouse model. Int. J. Mol. Sci. 20 (9), 2134. doi: 10.3390/ijms20092134 31052177 PMC6540607

[B175] Scheltens P. De Strooper B. Kivipelto M. Holstege H. Chételat G. Teunissen C. E.. (2021). Alzheimer’s disease. Lancet 397 (10284), 1577–1590. doi: 10.1016/s0140-6736(20)32205-4 33667416 PMC8354300

[B176] Sherwin E. Sandhu K. V. Dinan T. G. Cryan J. F. (2016). May the force be with you: the light and dark sides of the microbiota-Gut-Brain axis in neuropsychiatry. CNS Drugs 30 (11), 1019–1041. doi: 10.1007/s40263-016-0370-3 27417321 PMC5078156

[B177] Shin N. R. Lee J. C. Lee H. Y. Kim M. S. Whon T. W. Lee M. S.. (2014). An increase in the Akkermansia spp. population induced by metformin treatment improves glucose homeostasis in diet-induced obese mice. Gut 63 (5), 727–735. doi: 10.1136/gutjnl-2012-303839 23804561

[B178] Shin J. Noh J. R. Chang D. H. Kim Y. H. Kim M. H. Lee E. S.. (2019). Elucidation of Akkermansia muciniphila probiotic traits driven by mucin depletion. Front. Microbiol. 10. doi: 10.3389/fmicb.2019.01137 PMC6538878 31178843

[B179] Si J. Kang H. You H. J. Ko G. (2022). Revisiting the role of Akkermansia muciniphila as a therapeutic bacterium. Gut Microbes 14 (1), 2078619. doi: 10.1080/19490976.2022.2078619 35613313 PMC9135416

[B180] Singh V. Roth S. Llovera G. Sadler R. Garzetti D. Stecher B.. (2016). Microbiota dysbiosis controls the neuroinflammatory response after stroke. J. Neurosci. 36 (28), 7428–7440. doi: 10.1523/jneurosci.1114-16.2016 27413153 PMC6705544

[B181] Smith K. (2014). Mental health: a world of depression. Nature 515 (7526), 181. doi: 10.1038/515180a 25391942

[B182] Smith L. Schapira A. H. V. (2022). GBA variants and Parkinson disease: mechanisms and treatments. Cells 11 (8), 1261. doi: 10.3390/cells11081261 35455941 PMC9029385

[B183] Song J. (2023). Amygdala activity and amygdala-hippocampus connectivity: metabolic diseases, dementia, and neuropsychiatric issues. BioMed. Pharmacother. 162, 114647. doi: 10.1016/j.biopha.2023.114647 37011482

[B184] Stanley D. Mason L. J. Mackin K. E. Srikhanta Y. N. Lyras D. Prakash M. D.. (2016). Translocation and dissemination of commensal bacteria in post-stroke infection. Nat. Med. 22 (11), 1277–1284. doi: 10.1038/nm.4194 27694934

[B185] Stanley D. Moore R. J. Wong C. H. Y. (2018). An insight into intestinal mucosal microbiota disruption after stroke. Sci. Rep. 8 (1), 568. doi: 10.1038/s41598-017-18904-8 29330443 PMC5766598

[B186] Stefanis L. (2012). Alpha-synuclein in parkinson’s disease. Cold Spring Harb. Perspect. Med. 2 (2), a009399. doi: 10.1101/cshperspect.a009399 22355802 PMC3281589

[B187] Strati F. Cavalieri D. Albanese D. De Felice C. Donati C. Hayek J.. (2017). New evidences on the altered gut microbiota in autism spectrum disorders. Microbiome 5 (1), 24. doi: 10.1186/s40168-017-0242-1 28222761 PMC5320696

[B188] Sun Y. Zhu H. Cheng R. Tang Z. Zhang M. (2023). Outer membrane protein Amuc_1100 of Akkermansia muciniphila alleviates antibiotic-induced anxiety and depression-like behavior in mice. Physiol. Behav. 258, 114023. doi: 10.1016/j.physbeh.2022.114023 36336146

[B189] Takebayashi N. Maeshima H. Baba H. Nakano Y. Satomura E. Kita Y.. (2012). Duration of last depressive episode may influence serum BDNF levels in remitted patients with major depression. Depress Anxiety 29 (9), 775–779. doi: 10.1002/da.21933 22447660

[B190] Takewaki D. Suda W. Sato W. Takayasu L. Kumar N. Kimura K.. (2020). Alterations of the gut ecological and functional microenvironment in different stages of multiple sclerosis. Proc. Natl. Acad. Sci. U.S.A. 117 (36), 22402–22412. doi: 10.1073/pnas.2011703117 32839304 PMC7486801

[B191] Tan C. Wu Q. Wang H. Gao X. Xu R. Cui Z.. (2021). Dysbiosis of gut microbiota and short-chain fatty acids in acute ischemic stroke and the subsequent risk for poor functional outcomes. JPEN J. Parenter Enteral Nutr. 45 (3), 518–529. doi: 10.1002/jpen.1861 32473086 PMC8048557

[B192] Tankou S. K. Regev K. Healy B. C. Tjon E. Laghi L. Cox L. M.. (2018). A probiotic modulates the microbiome and immunity in multiple sclerosis. Ann. Neurol. 83 (6), 1147–1161. doi: 10.1002/ana.25244 29679417 PMC6181139

[B193] Tundo A. De Filippis R. Proietti L. (2015). Pharmacologic approaches to treatment resistant depression: evidences and personal experience. World J. Psychiatry 5 (3), 330–341. doi: 10.5498/wjp.v5.i3.330 26425446 PMC4582308

[B194] Tuomola E. Crittenden R. Playne M. Isolauri E. Salminen S. (2001). Quality assurance criteria for probiotic bacteria. Am. J. Clin. Nutr. 73 (2 Suppl), 393s–398s. doi: 10.1093/ajcn/73.2.393s 11157347

[B195] Ulamek-Koziol M. Czuczwar S. J. Januszewski S. Pluta R. (2020). Proteomic and genomic changes in tau protein, which are associated with alzheimer’s disease after ischemia-reperfusion brain injury. Int. J. Mol. Sci. 21 (3), 892. doi: 10.3390/ijms21030892 32019137 PMC7037789

[B196] Valentino F. Bruno L. P. Doddato G. Giliberti A. Tita R. Resciniti S.. (2021). Exome sequencing in 200 intellectual Disability/Autistic patients: new candidates and atypical presentations. Brain Sci. 11 (7), 936. doi: 10.3390/brainsci11070936 34356170 PMC8303733

[B197] Van Der Lugt B. Van Beek A. A. Aalvink S. Meijer B. Sovran B. Vermeij W. P.. (2019). Akkermansia muciniphila ameliorates the age-related decline in colonic mucus thickness and attenuates immune activation in accelerated aging Ercc1 (-/Δ7) mice. Immun. Ageing 16, 6. doi: 10.1186/s12979-019-0145-z 30899315 PMC6408808

[B198] Van Passel M. W. Kant R. Zoetendal E. G. Plugge C. M. Derrien M. Malfatti S. A.. (2011). The genome of Akkermansia muciniphila, a dedicated intestinal mucin degrader, and its use in exploring intestinal metagenomes. PloS One 6 (3), e16876. doi: 10.1371/journal.pone.0016876 21390229 PMC3048395

[B199] Vicentini F. A. Keenan C. M. Wallace L. E. Woods C. Cavin J. B. Flockton A. R.. (2021). Intestinal microbiota shapes gut physiology and regulates enteric neurons and glia. Microbiome 9 (1), 210. doi: 10.1186/s40168-021-01165-z 34702353 PMC8549243

[B200] Wang F. Cai K. Xiao Q. He L. Xie L. Liu Z. (2022). Akkermansia muciniphila administration exacerbated the development of colitis-associated colorectal cancer in mice. J. Cancer 13 (1), 124–133. doi: 10.7150/jca.63578 34976176 PMC8692691

[B201] Wang L. Christophersen C. T. Sorich M. J. Gerber J. P. Angley M. T. Conlon M. A. (2011). Low relative abundances of the mucolytic bacterium Akkermansia muciniphila and bifidobacterium spp. in feces of children with autism. Appl. Environ. Microbiol. 77 (18), 6718–6721. doi: 10.1128/AEM.05212-11 21784919 PMC3187122

[B202] Wang Y. Li L. Zhao X. Sui S. Wang Q. Shi G.. (2022). Intestinal microflora changes in patients with mild alzheimer’s disease in a Chinese cohort. J. Alzheimers Dis. 88 (2), 563–575. doi: 10.3233/JAD-220076 35662119

[B203] Wang L. Tang L. Feng Y. Zhao S. Han M. Zhang C.. (2020). A purified membrane protein from Akkermansia muciniphila or the pasteurised bacterium blunts colitis associated tumourigenesis by modulation of CD8(+) T cells in mice. Gut 69 (11), 1988–1997. doi: 10.1136/gutjnl-2019-320105 32169907 PMC7569398

[B204] Wang M. Wan J. Rong H. He F. Wang H. Zhou J.. (2019). Alterations in gut glutamate metabolism associated with changes in gut microbiota composition in children with autism spectrum disorder. mSystems 4 (1), e00321–e00318. doi: 10.1128/mSystems.00321-18 30701194 PMC6351726

[B205] Wang J. Xu W. Wang R. Cheng R. Tang Z. Zhang M. (2021). The outer membrane protein Amuc_1100 of Akkermansia muciniphila promotes intestinal 5-HT biosynthesis and extracellular availability through TLR2 signalling. Food Funct. 12 (8), 3597–3610. doi: 10.1039/d1fo00115a 33900345

[B206] Weir T. L. Manter D. K. Sheflin A. M. Barnett B. A. Heuberger A. L. Ryan E. P. (2013). Stool microbiome and metabolome differences between colorectal cancer patients and healthy adults. PloS One 8 (8), e70803. doi: 10.1371/journal.pone.0070803 23940645 PMC3735522

[B207] Wu W. Lv L. Shi D. Ye J. Fang D. Guo F.. (2017). Protective effect of Akkermansia muciniphila against immune-mediated liver injury in a mouse model. Front. Microbiol. 8. doi: 10.3389/fmicb.2017.01804 PMC5626943 29033903

[B208] Wu G. Zhang C. Wu H. Wang R. Shen J. Wang L.. (2017). Genomic microdiversity of bifidobacterium pseudocatenulatum underlying differential strain-level responses to dietary carbohydrate intervention. mBio 8 (1), e02348–e02316. doi: 10.1128/mBio.02348-16 28196965 PMC5312088

[B209] Xia J. Lv L. Liu B. (2022). Akkermansia muciniphila ameliorates acetaminophen-induced liver injury by regulating gut microbial composition and metabolism. Microbiol. Spectr. 10 (1), e0159621. doi: 10.1128/spectrum.01596-21 35107323 PMC8809353

[B210] Xiang L. Lou Y. Liu L. Liu Y. Zhang W. Deng J.. (2020). Gut microbiotic features aiding the diagnosis of acute ischemic stroke. Front. Cell Infect. Microbiol. 10. doi: 10.3389/fcimb.2020.587284 PMC7779606 33409158

[B211] Xin Y. Diling C. Jian Y. Ting L. Guoyan H. Hualun L.. (2018). Effects of oligosaccharides from morinda officinalis on gut microbiota and metabolome of APP/PS1 transgenic mice. Front. Neurol. 9. doi: 10.3389/fneur.2018.00412 PMC6013575 29962999

[B212] Xu Z. Zhao L. Yin L. Liu Y. Ren Y. Yang G.. (2022). MRI-Based machine learning model: a potential modality for predicting cognitive dysfunction in patients with type 2 diabetes mellitus. Front. Bioeng Biotechnol. 10. doi: 10.3389/fbioe.2022.1082794 PMC9725113 36483770

[B213] Yaghoubfar R. Behrouzi A. Ashrafian F. Shahryari A. Moradi H. R. Choopani S.. (2020). Modulation of serotonin signaling/metabolism by Akkermansia muciniphila and its extracellular vesicles through the gut-brain axis in mice. Sci. Rep. 10 (1), 22119. doi: 10.1038/s41598-020-79171-8 33335202 PMC7747642

[B214] Yang Y. Zhong Z. Wang B. Xia X. Yao W. Huang L.. (2019). Early-life high-fat diet-induced obesity programs hippocampal development and cognitive functions via regulation of gut commensal Akkermansia muciniphila. Neuropsychopharmacology 44 (12), 2054–2064. doi: 10.1038/s41386-019-0437-1 31207607 PMC6897910

[B215] Yao Y. Cai X. Fei W. Ye Y. Zhao M. Zheng C. (2022). The role of short-chain fatty acids in immunity, inflammation and metabolism. Crit. Rev. Food Sci. Nutr. 62 (1), 1–12. doi: 10.1080/10408398.2020.1854675 33261516

[B216] Yao S. Xie H. Wang Y. Shen N. Chen Q. Zhao Y.. (2023). Predictive microbial feature analysis in patients with depression after acute ischemic stroke. Front. Aging Neurosci. 15. doi: 10.3389/fnagi.2023.1116065 PMC10076592 37032826

[B217] Yohn C. N. Gergues M. M. Samuels B. A. (2017). The role of 5-HT receptors in depression. Mol. Brain 10 (1), 28. doi: 10.1186/s13041-017-0306-y 28646910 PMC5483313

[B218] Yoon H. S. Cho C. H. Yun M. S. Jang S. J. You H. J. Kim J. H.. (2021). Akkermansia muciniphila secretes a glucagon-like peptide-1-inducing protein that improves glucose homeostasis and ameliorates metabolic disease in mice. Nat. Microbiol. 6 (5), 563–573. doi: 10.1038/s41564-021-00880-5 33820962

[B219] Yu J. H. Han K. Park S. Cho H. Lee D. Y. Kim J. W.. (2020). Incidence and risk factors for dementia in type 2 diabetes mellitus: a nationwide population-based study in Korea. Diabetes Metab. J. 44 (1), 113–124. doi: 10.4093/dmj.2018.0216 31769236 PMC7043975

[B220] Yu J. Liu T. Gao Z. Liu R. Wang Z. Chen Y.. (2022). Akkermansia muciniphila colonization alleviating high fructose and restraint stress-induced jejunal mucosal barrier disruption. Nutrients 14 (15), 3164. doi: 10.3390/nu14153164 35956340 PMC9370786

[B221] Zapała B. Stefura T. Wójcik-Pędziwiatr M. Kabut R. Bałajewicz-Nowak M. Milewicz T.. (2021). Differences in the composition of gut microbiota between patients with parkinson’s disease and healthy controls: a cohort study. J. Clin. Med. 10 (23), 5698. doi: 10.3390/jcm10235698 34884399 PMC8658639

[B222] Zhai Q. Feng S. Arjan N. Chen W. (2019). A next generation probiotic, Akkermansia muciniphila. Crit. Rev. Food Sci. Nutr. 59 (19), 3227–3236. doi: 10.1080/10408398.2018.1517725 30373382

[B223] Zhang T. Li Q. Cheng L. Buch H. Zhang F. (2019). Akkermansia muciniphila is a promising probiotic. Microb. Biotechnol. 12 (6), 1109–1125. doi: 10.1111/1751-7915.13410 31006995 PMC6801136

[B224] Zhang J. Ni Y. Qian L. Fang Q. Zheng T. Zhang M.. (2021). Decreased abundance of Akkermansia muciniphila leads to the impairment of insulin secretion and glucose homeostasis in lean type 2 diabetes. Adv. Sci. (Weinh) 8 (16), e2100536. doi: 10.1002/advs.202100536 34085773 PMC8373164

[B225] Zhao S. Liu W. Wang J. Shi J. Sun Y. Wang W.. (2017). Akkermansia muciniphila improves metabolic profiles by reducing inflammation in chow diet-fed mice. J. Mol. Endocrinol. 58 (1), 1–14. doi: 10.1530/jme-16-0054 27821438

[B226] Zhao Q. Yu J. Hao Y. Zhou H. Hu Y. Zhang C.. (2023). Akkermansia muciniphila plays critical roles in host health. Crit. Rev. Microbiol. 49 (1), 82–100. doi: 10.1080/1040841x.2022.2037506 35603929

[B227] Zheng B. C. L. Cantley (2007). Regulation of epithelial tight junction assembly anddisassembly by AMP-activated protein kinase. Proc. Natl. Acad. Sci. U.S.A. 104, 819–822. doi: 10.1073/pnas.0610157104 17204563 PMC1783397

[B228] Zheng M. Han R. Yuan Y. Xing Y. Zhang W. Sun Z.. (2022). The role of Akkermansia muciniphila in inflammatory bowel disease: current knowledge and perspectives. Front. Immunol. 13. doi: 10.3389/fimmu.2022.1089600 PMC9853388 36685588

[B229] Zhong D. Y. Li L. Ma R. M. Deng Y. H. (2021). The effect of probiotics in stroke treatment. Evid Based Complement Alternat Med. 2021, 4877311. doi: 10.1155/2021/4877311 34745285 PMC8568545

[B230] Zhuang Z. Q. Shen L. L. Li W. W. Fu X. Zeng F. Gui L.. (2018). Gut microbiota is altered in patients with alzheimer’s disease. J. Alzheimers Dis. 63 (4), 1337–1346. doi: 10.3233/jad-180176 29758946

[B231] Zou R. Xu F. Wang Y. Duan M. Guo M. Zhang Q.. (2020). Changes in the gut microbiota of children with autism spectrum disorder. Autism Res. 13 (9), 1614–1625. doi: 10.1002/aur.2358 32830918

